# Human Enzyme PADI4 Binds to the Nuclear Carrier Importin α3

**DOI:** 10.3390/cells11142166

**Published:** 2022-07-11

**Authors:** José L. Neira, Bruno Rizzuti, Olga Abián, Salomé Araujo-Abad, Adrián Velázquez-Campoy, Camino de Juan Romero

**Affiliations:** 1 Instituto de Investigación, Desarrollo e Innovación en Biotecnología Sanitaria de Elche, Universidad Miguel Hernández, 03202 Elche, Spain; lourdes.araujo@goumh.umh.es (S.A.-A.); m.juan@umh.es (C.d.J.R.); 2 Instituto de Biocomputación y Física de Sistemas Complejos–Unidad mixta GBsC-CSIC-BIFI, Universidad de Zaragoza, 50018 Zaragoza, Spain; oabifra@unizar.es (O.A.); adrianvc@unizar.es (A.V.-C.); 3 CNR-NANOTEC, SS Rende (CS), Department of Physics, University of Calabria, 87036 Rende, Italy; 4 Instituto de Investigación Sanitaria Aragón (IIS Aragón), 50009 Zaragoza, Spain; 5 Centro de Investigación Biomédica en Red en el Área Temática de Enfermedades Hepáticas y Digestivas (CIBERehd), 28029 Madrid, Spain; 6 Departamento de Bioquímica y Biología Molecular y Celular, Universidad de Zaragoza, 50009 Zaragoza, Spain; 7 Centro de Biotecnología, Universidad Nacional de Loja, Avda. Pío Jaramillo Alvarado s/n, Loja 110111, Ecuador; 8 Unidad de Investigación, Fundación para el Fomento de la Investigación Sanitaria y Biomédica de la Comunidad Valenciana (FISABIO), Hospital General Universitario de Elche, Camí de l’Almazara 11, 03203 Elche, Spain

**Keywords:** PADI4, nuclear localization signal, binding, calorimetry, fluorescence, molecular docking, cancer

## Abstract

PADI4 is a peptidyl-arginine deiminase (PADI) involved in the conversion of arginine to citrulline. PADI4 is present in macrophages, monocytes, granulocytes, and several cancer cells. It is the only PADI family member observed within both the nucleus and the cytoplasm. PADI4 has a predicted nuclear localization sequence (NLS) comprising residues Pro56 to Ser83, to allow for nuclear translocation. Recent predictors also suggest that the region Arg495 to Ile526 is a possible NLS. To understand how PADI4 is involved in cancer, we studied the ability of intact PADI4 to bind importin α3 (Impα3), a nuclear transport factor that plays tumor-promoting roles in several cancers, and its truncated species (ΔImpα3) without the importin-binding domain (IBB), by using fluorescence, circular dichroism (CD), and isothermal titration calorimetry (ITC). Furthermore, the binding of two peptides, encompassing the first and the second NLS regions, was also studied using the same methods and molecular docking simulations. PADI4 interacted with both importin species, with affinity constants of ~1–5 µM. The isolated peptides also interacted with both importins. The molecular simulations predict that the anchoring of both peptides takes place in the major binding site of Impα3 for the NLS of cargo proteins. These findings suggest that both NLS regions were essentially responsible for the binding of PADI4 to the two importin species. Our data are discussed within the framework of a cell mechanism of nuclear transport that is crucial in cancer.

## 1. Introduction

Deamination, or citrullination, is a post-translational modification (PTM) catalyzed by l-arginine iminohydrolases (PADIs), also known as peptidyl-arginine deiminases (EC 3.5.3.15). PADIs have key roles in nerve growth, development of embryos, trauma apoptosis, aging in tissues, epithelial terminal differentiation, and transcriptional regulation of gene expression [1,2,3,4,5,6,7,8]. Moreover, several maladies such as rheumatoid arthritis, Alzheimer’s disease, psoriasis, multiple sclerosis, and many types of cancers are associated with the increased presence of PADIs and their citrullinated targets [7,9,10,11].

PADI1, PADI2, PADI3, PADI4, and PADI6 are the five human isozymes [12,13,14,15,16,17,18], each having a tissue-specific expression. An increase of enzyme activity is observed for several PADI4 haplotype mutants during the apoptosis enhanced through the mitochondrial pathway [19]. Furthermore, PADI4 is involved in the expression of p53 target genes, as well as in the gene expression of p53 [20,21].

PADI4, as well as PAD2 under some conditions, has been detected in both the cytoplasm and the nucleus [22,23,24], but the remaining isoforms are found in the cytoplasm. Because some of the PADI4 functions are carried out inside the nucleus, the protein must be translocated through the nuclear pore complex (NPC). PADI4 is involved in the citrullination of histones H1, H2A, H3, and H4, where a competitive inhibition between histone methylation and citrullination takes place, resulting in cancer development and progression [25]. Furthermore, citrullination also competes with histone deacetylation to regulate cancer growth and evolution [25]. Lastly, within the nucleus, p53 binds to histone deacetylase 2 and PADI4 through distinct domains, thus regulating PADI4-mediated histone citrullination. All these functions are carried out inside the nucleus and, therefore, require prior translocation of PADI4.

Nuclear translocation generally occurs through importins, together with other auxiliary proteins [26,27]. The classical nuclear import pathway is triggered by the recognition of a nuclear localization signal (NLS) polypeptide patch in the cargo by importin α [26]. The different types of NLSs, their ways of anchoring to their target, and their structures in isolation or when bound to transport factors have been extensively reviewed [28]. The cargo–importin α complex then binds to importin β, and the so-formed complex of the three proteins moves through the NPC. Importin α is a modular protein with several α-helix repeat armadillo (ARM) units [26,27]. It has two domains: (i) a 60-residue-long importin β-binding (IBB) domain, located at the N-terminal region, which is used for binding to importin β before transport through the NPC, and (ii) an NLS-binding motif formed by 10 ARM units, located at the C-terminus [29]. In the absence of importin β, the IBB domain, which mimics an NLS, occupies the ARM regions implicated in NLS recognition [29]. This intramolecular interaction has an auto-inhibitory function [29]. Variations in the nuclear transport through the importin route are important events regulating gene expression, signal transduction, and cell-cycle regulation; therefore, they can play a key role in cancer development and cell transformation [30,31,32]. As an example, abnormal overexpression of importin α1 has been observed in hepatocellular carcinoma [33]. Aberrant nuclear translocation is one of the hallmark features of cancer [32], also making the proteins at play in such a translocation process potential therapeutic cancer targets.

Because of the importance of the nuclear translocation in PADI4 functions, we decided to study its interaction with human importin α3 (Impα3), also called KPNA4, and with its truncated species lacking the IBB domain (ΔImpα3). Impα3 has been reported to be associated with multiple cancers (such as glioblastoma, prostate cancer, hepatocellular carcinoma, lung cancer, and ovarian cancer). Impα3 promotes tumor proliferation by facilitating several cancer-related processes [32,34,35,36,37,38]. We considered Impα3 as a target for PADI4 because (i) it is largely conserved among different species [39], and (ii) it has increased flexibility compared with other importins, as concluded by the structural B-factors from X-ray data; this feature confers this importin isoform a greater ability to interact with various cargos [40]. From an experimental point of view, Impα3 can also be easily expressed and purified for in vitro structural and binding studies [40,41,42]. In addition, Impα3 can be considered a model protein to investigate how the NLS sequence of the cargo can affect the thermodynamic parameters in the binding process, and we have already carried out several studies of the binding of Impα3 with other NLSs which can be used as a comparison [41,42,43]. Lastly, by studying both importin species (with and without the IBB), we could explore whether the absence of the IBB domain affects the binding of the peptide encompassing the NLS region, as studied in the case of other NLSs of several proteins (see [41,42,43] and references therein).

In our experiments, we firstly explored the binding between intact PADI4 and either Impα3 or ΔImpα3. Next, we described the binding of the two predicted NLS regions of PADI4 (NLS1-PADI4 and NLS2-PADI4) to the two importin species. Fluorescence, CD, and ITC confirmed that the binding took place between the intact PADI4 and the two importin species. On the other hand, we found that the two peptides corresponding to the isolated NLS1-PADI4 and NLS2-PADI4 sequences, which were mainly disordered in solution, were capable of binding to both importin species, as tested by fluorescence, ITC, and BLI. Moreover, molecular docking simulations suggested that the core regions of NLS1-PADI4 and NLS2-PADI4 were responsible for the binding, and they were both capable of anchoring to the major binding site for the NLSs of cargo proteins to Impα3. Taken together, our in vitro and in silico results suggest that PADI4 requires Impα3 to be translocated into the nucleus, and this interaction is mediated by two possible regions located at either terminus of the cargo protein. Given the importance of PADI4 in the development of tumor cells and the involvement of importins in such processes, our results can provide a molecular description of the basic binding mechanism that may lead to cancer progression.

## 2. Materials and Methods

### 2.1. Materials

The same materials used in this study have been described previously [41,42,43].

### 2.2. Protein Expression and Purification

PADI4, Impα3, and ΔImpα3 were purified as previously described [24,41,42,43]. The concentrations of the proteins were calculated by UV absorbance, using an extinction coefficient at 280 nm; this parameter was estimated from the number of tyrosines and tryptophans in each of these proteins [44]. In the remainder of the paper, PADI4 protein concentrations are expressed as protomer concentrations.

### 2.3. Prediction and Synthesis of the NLS Regions of PADI4

The NLS regions for the PADI4 sequence were predicted using the web server cNLS Mapper [45,46], available at http://nls-mapper.iab.keio.ac.jp (accessed 8 December 2021). The results pointed out the occurrence of two possible NLS regions. The one with the lowest score (5.3, in arbitrary units), hereafter indicated as NLS1, overlapped with that already predicted as the canonical NLS region [47], with the sequence P^56^PAKKKSTGSSTWPLDPGVEVTLTMKVASGS^86^ (according to the numbering of the intact PADI4). The predicted region with the highest score (6.1) had the sequence R^495^SCYKLFQEQQNEGHGEALLFEGIKKKKQQKI^526^, which was indicated as NLS2. These two regions were synthesized as isolated peptides NLS1-PADI4 (residues Ala58–Ser86) and NLS2-PADI4 (residues Tyr498–Ile526), both slightly shorter than the predicted NLS regions to avoid potential complications due the presence of some residues (Pro56/Pro57 for NLS1 and Cys497 for NLS2) at one of their termini. The peptides were also acetylated and amidated at the N- and C-termini, respectively, to avoid fraying effects. The two peptides NLS1-PADI4 and NLS2-PADI4 were produced by Genscript (Leiden, Netherlands) and NZYtech (Lisbon, Portugal), respectively, with a purity larger than 95%. Peptide concentrations were determined from the absorbance of either Tyr498 (NLS2-PADI4) or Trp68 (NLS1-PADI4) [44].

### 2.4. Fluorescence

#### 2.4.1. Steady-State Fluorescence

Spectra were collected on a Cary Varian spectrofluorometer (Agilent, Santa Clara, CA, USA), interfaced with a Peltier unit. Following the standard protocols used in our laboratories, the samples were prepared the day before and left overnight at 5 °C; before experiments, samples were left for 1 h at 25 °C, where experiments were acquired. A 1 cm pathlength quartz cell (Hellma, Kruibeke, Belgium) was used. The concentration of PADI4 or NLS1/2-PADI4 peptides was 20 µM, and that of each importin species was 2 µM. Samples containing the corresponding isolated peptides, the isolated PADI4, the isolated corresponding importin species, and the corresponding mixtures (at the concentrations indicated above) were prepared. Experiments were performed with samples in 50 mM sodium phosphate buffer, pH 7.0. Fluorescence experiments were repeated in triplicates with newly prepared samples. Variations of results among the experiments were lower than 5%.

Polypeptide samples were excited either at 280 or 295 nm (although NLS2-PADI4 has only a single tyrosine). The other experimental parameters used in the experiments have been described elsewhere [48]. Appropriate blank corrections were made in all spectra.

#### 2.4.2. Binding Experiments with PADI4

For the titration between either Impα3 or ΔImpα3 and intact PADI4, increasing amounts of the corresponding importin species, in the concentration range 0–25 µM, were added to a solution with a fixed concentration of intact PADI4 (3 µM). Experiments were carried out in 20 mM Tris buffer (pH 7.5), 5 mM TCEP, 150 mM NaCl, and 5% glycerol at 25 °C (the storage buffer of PADI4). The experimental setup was the same as in the steady-state fluorescence experiments. Blank corrections containing the amount of each importin species were subtracted. Inner-filter effects were corrected [49]. Each titration (Impα3 with PADI4 or ΔImpα3 with PADI4) was repeated three times, using new samples. In the three cases, the variations in the results were lower than 10%.

Handling and preparation of samples were the same described in Section 2.4.1. The dissociation constant of the corresponding complex, *K_d_*, was calculated by fitting the binding isotherm to the general binding model, explicitly considering ligand depletion [50,51].

(1) $$\begin{array}{c} F=F_{0}+\frac{\Delta F_{max}}{2{\left[PADI4\right]}_{T}}({\left[Imp\alpha 3_{species}\right]}_{T}+{\left[PADI4\right]}_{T}+K_{d})-\\ \sqrt{\left({\left({\left[Imp\alpha 3_{species}\right]}_{T}+{\left[PADI4\right]}_{T}+K_{d}\right)}^{2}-4{\left[Imp\alpha 3_{species}\right]}_{T}{\left[PADI4\right]}_{T}\right)}, \end{array}$$

where *F* is the measured fluorescence at any particular concentration of importin species after subtraction of the matching blank concentration of importin species, Δ*F_max_* is the largest change in the fluorescence of importin species when all polypeptide molecules were bound, compared to the fluorescence of each unbound chain, *F*_0_ is the fluorescence intensity when no importin species were added, [*PADI*4]_T_ is the constant, total concentration of PADI4 (3 µM), and [*Impα*3*_species_*]_T_ is that of either Impα3 or ΔImpα3, which was varied during the titration. Fitting to Equation (1) was carried out using KaleidaGraph (Synergy software, Reading, PA, USA).

#### 2.4.3. Binding Experiments with NLS1/2-PADI4

For the titration between either Impα3 or ΔImpα3 and NLS1/2-PADI4, increasing amounts of the corresponding peptide, in the concentration range 0–20 µM, were added to a solution with a fixed concentration of either Impα3 or ΔImpα3 (3 µM). Experiments were carried out in the same buffer used for the titration of the intact PADI4 at 25 °C. The experimental setup was the same as in the steady-state fluorescence experiments. Blank corrections were subtracted in all cases. Spectra were corrected for inner-filter effects during fluorescence excitation [49]. Each titration (Impα3 with NLS1/2-PADI4 or ΔImpα3 with NLS1/2-PADI4) was repeated three times, using newly prepared samples. In the three cases, the variations in the results were lower than 10%.

Handling and preparation of samples were the same described in Section 2.4.1. The dissociation constant for each complex, *K_d_*, was calculated by fitting the binding isotherm constructed to the general binding model, explicitly considering ligand depletion [50,51].

(2) $$\begin{array}{c} F=F_{0}+\frac{\Delta F_{max}}{2{\left[Imp\alpha 3_{species}\right]}_{T}}({\left[Imp\alpha 3_{species}\right]}_{T}+{\left[NLS1/2_{PADI4}\right]}_{T}+K_{d})\\ \;\;\;\;\;\;\;\;\;\;\;\;\;\;\;\;\;\;-\sqrt{\left({\left({\left[Imp\alpha 3_{species}\right]}_{T}+{\left[NLS1/2_{PADI4}\right]}_{T}+K_{d}\right)}^{2}-4{\left[Imp\alpha 3_{species}\right]}_{T}{\left[NLS1/2_{PADI4}\right]}_{T}\right)}, \end{array}$$

where *F* is the measured fluorescence at any particular concentration of the corresponding peptide after subtraction of the matching blank concentration of NLS1/2-PADI4, Δ*F_max_* is the largest change in the fluorescence of the corresponding peptide when all polypeptide molecules were forming the complex, compared to the fluorescence of each isolated chain, *F*_0_ is the fluorescence intensity when no NLS1/2-PADI4 was added, [NLS1/2_PADI4_]*_T_* is the total concentration of the corresponding peptide, which was varied during the titration, and [*Impα*3*_species_*]*_T_* is that of either Impα3 or ΔImpα3, which was kept constant during the titration. Fitting to Equation (2) was carried out using KaleidaGraph (Synergy software, Reading, PA, USA).

### 2.5. Circular Dichroism (CD)

Far-UV CD spectra were collected on a Jasco J810 spectropolarimeter (Jasco, Tokyo, Japan) interfaced with a Peltier unit. The instrument was periodically calibrated with (+)-10-camphorsulfonic acid. A cell of path length 0.1 cm was used (Hellma, Kruibeke, Belgium). All spectra were corrected by subtracting the corresponding baseline. Concentration of each polypeptide (importin species and either NLS1/2-PADI4 or PADI4) and the buffers were the same used in the fluorescence experiments (Section 2.4).

Isothermal wavelength spectra of each isolated macromolecule and that of the complex were acquired as an average of six scans, at a scan speed of 50 nm/min, with a response time of 2 s and a bandwidth of 1 nm. Handling and preparation of samples were the same described in Section 2.4.1.

### 2.6. Nuclear Magnetic Resonance (NMR) Spectroscopy

The NMR spectra were acquired at 10 °C on a Bruker Avance 500 MHz spectrometer (Bruker GmbH, Karlsruhe, Germany), equipped with a triple resonance probe and z-pulse field gradients. Spectra were processed with Bruker TopSpin 2.1 (Bruker GmbH, Karlsruhe, Germany). All NMR experiments with NLS1/2-PADI4 peptides were carried out in 100 mM sodium phosphate buffer (not corrected for isotope effects), pH 7.0. Spectra were calibrated with TSP, by considering pH-dependent changes of its chemical shifts [52]; probe temperature was calibrated with pure methanol [52].

#### 2.6.1. 1D-^1^H-NMR Spectra

A total of 48 scans were acquired with 16 K acquisition points for the homonuclear 1D-^1^H-NMR spectra of each isolated peptide at a concentration of 1.2 mM. The water signal was suppressed with the WATERGATE sequence [53]. The spectra were processed by using TopSpin 2.1 with an exponential window, after zero-filling.

#### 2.6.2. Translational Diffusion NMR (DOSY)

The NLS1/2-PADI4 concentration in DOSY experiments was 100 µM, and 128 scans were acquired, where the gradient strength was varied linearly. Details on the experimental conditions and fitting of the resulting curves have been described elsewhere [48]. A final concentration of 1% of dioxane, with an assumed hydrodynamic radius, *R*_h_, of 2.12 Å [54], was added to the solution.

#### 2.6.3. 2D-^1^H-NMR Spectra

Two-dimensional spectra of NLS2-PADI4 (at 1.2 mM) were acquired in each dimension in phase-sensitive mode by using the time-proportional phase incrementation technique [55] and a spectral width of 5500 Hz. Standard TOCSY (mixing time of 80 ms) [56] and NOESY experiments (a mixing time of 250 ms) [56,57,58], with the WATERGATE sequence [53], as well as experimental, processing, and assigning details, were the same used in acquiring, processing, and analyzing the spectra of other NLSs [41,42,43]. The chemical shift values of H_α_ protons in random-coil regions were obtained from tabulated data, corrected by neighboring residue effects [59,60,61].

### 2.7. Isothermal Titration Calorimetry (ITC)

Calorimetric titrations for testing the interaction of PADI4, as well as for the interaction of NLS1/2-PADI4 peptides, with both importins, Impα3 and ΔImpα3, were carried out in an Auto-iTC200 automated high-sensitivity calorimeter (MicroCal, Malvern-Panalytical, Malvern, UK). Experiments were performed in 20 mM Tris buffer (pH 7.5), 5 mM TCEP, 150 mM NaCl, and 5% glycerol at 25 °C. PADI4 or the peptide solution (100 μM) in the injection syringe was titrated into the importin solution (10 μM) in the calorimetric cell. The remaining experimental and processing details have been described previously [41,42,43]. Due to the presence of glycerol in solution, background injection (included as an adjustable parameter in data fitting) was rather large. The data analysis was conducted in Origin 7.0 (OriginLab, Northampton, MA, USA) with user-defined fitting functions.

### 2.8. Biolayer Inteferometry (BLI)

#### 2.8.1. Experimental Design

The association (*k_on_*) and dissociation (*k_off_*) rate constants of the binding of NLS1/2-PADI4 peptides to Impα3 or ΔImpα3 were determined using a BLItz system (ForteBio, Pall, Barcelona, Spain) [62]. The buffer used in the experiments was that recommended by the manufacturer. Since Impα3 and ΔImpα3 had a His-tag, they were immobilized on His-tag biosensors (Forte Bio) at 0.3 µM. The peptide concentrations were in the range from 1 to 7 µM during the association step. The general scheme of the protein association/dissociation reactions in the BLItz system for NLS1/2-PADI4 with Impα3 and ΔImpα3 immobilized on the biosensor was similar to that described previously [63].

#### 2.8.2. Fitting of the Sensorgrams

Fittings of the sensorgrams was carried out using KaleidaGraph (Synergy software, Reading, PA, USA) [63]. The interferometry response during the association step, *R(t)* (measured in response units, RU), and the binding rate, *dR(t)*/*dt*, can be used to evaluate the kinetics of the formation of the Impα3/ΔImpα3–NLS1/2-PADI4 complex, according to

(3) $$\frac{dR}{dt}=k_{on}\left[NLS1/2_{PADI4}\right]\left(R_{max}-R\left(t\right)\right)-k_{off}R\left(t\right),$$

where *R_max_* is proportional to the total concentration of biosensor-bound importin species, and [NLS1/2PADI4] represents the concentration of the corresponding NLS1/2-PADI4 peptide.

In Equation (3), *R(t)* is given by

(4) $$R\left(t\right)=R_{eq}-R_{eq}e^{\left(-k_{obs}\;\left(t-t_{0}\right)\right)},$$

where *R_eq_* is the steady-state (or equilibrium) response obtained at infinite time, when *dR(t)*/*dt* = 0, and *t*_0_ = 180 s is the time at which the association step between biosensor-immobilized Impα3/ΔImpα3 and NLS1/2-PADI4 in the solution started. We fitted the experimentally obtained *R*(*t*) under any condition as

(5) $$R\left(t\right)=R_{eq}-R_{eq}e^{\left(-k_{obs}\;\left(t-t_{0}\right)\right)}-R_{eq}^{\prime }e^{\left(-k_{obs2}^{\prime }\;\left(t-t_{0}\right)\right)},$$

since an *F* statistical analysis test of the kinetic constants obtained with a fitting to Equations (4) or (5) was always better in the latter, two-exponential case (at 95% confidence level). With Equation (5), we are assuming that the equilibrium response at infinite time (that is, *R_eq_*) is reached with the fastest exponential. The largest-amplitude exponential had a concentration-dependent kinetic rate, and it was used for the pseudo-first-order plots, where the value of *k_obs_* is given by

(6) $$k_{obs}=k_{on}\;\left[NLS1/2_{PADI4}\right]+k_{off}.$$

The kinetic rate from the second exponential (with a total amplitude smaller than 5%, in all cases, and slower than the other phase) in Equation (5) remained constant at all the peptide concentrations explored.

The dissociation process was always fitted to a single exponential, with *R(t)* given by

(7) $$R\left(t\right)=R_{1}\;e^{\left(-k_{off}\left(t-t_{0}\right)\right)},$$

where *t*_0_ = 300 is the time at which the dissociation of the peptide from the biosensor-bound Impα3/ΔImpα3 started in our experimental setup, and *R*_1_ is the response level when dissociation starts.

### 2.9. Molecular Docking

Molecular simulations were performed using the docking software AutoDock Vina (version 1.1.2) [64], following a methodology we already employed for the virtual screening of other NLS peptides binding to Impα3 [41,42,43]. The protein was modeled in the IBB-depleted form on the basis of the Protein Data Bank (PDB) entry 5XZX [65], which reports the crystallographic complex with the NLS of the Ran-binding protein 3 anchored within the major binding site of Impα3. Further simulations were performed with the protein modelled on the basis of PDB entry 5X8N [66], which reports the complex of Impα1 with the NLS of the Epstein–Barr virus EBNA-LP protein bound within the same site. The ligand and the crystallographic waters were not considered as being part of the docking host in the simulations. The search region in the docking calculations (size: 50 Å × 90 Å × 90 Å) was centered on the protein and comprised its entire volume. All simulation runs were performed with very high exhaustiveness, 32 times larger than the default value [67].

The two NLS1/2-PADI4 peptides have a number of degrees of freedom that is too large (>100 rotatable dihedral angles) to be investigated in single docking experiments. Thus, the sequence of both peptides was divided into seven-residue fragments, each having five residues in common with the adjacent one and shifted by two residues. The fragments were capped by inserting a methyl group at both their N- and C-termini, except the last fragment for which the –NH_2_ terminal moiety was maintained. This modeling was adopted to avoid potential artefacts due to the introduction of polar hydrogens at the extremities of a fragment, which do not exist in the main chain of the peptides and of the native protein. The use of NLS fragments reduced the average number of degrees of freedom within the limit (≤32 rotatable dihedral angles) considered reliable for a successful use of AutoDock Vina [64]. The binding score assigned to each peptide residue was the average of the affinities obtained for all the seven-residue fragments that contained that specific amino acid.

The procedure described above was strictly followed for the peptide corresponding to NLS1-PADI4 (sequence: A^58^KKKSTGSSTWPLDPGVEVTLTMKVAS^84^), to obtain the eleven fragments A^58^KKKSTG^64^, K^60^KSTGSS^66^,…, L^78^TMKVAS^84^. For the NLS2-PADI4 (sequence: Y^498^KLFQEQQNEGHGEALLFEGIKKKKQQKI^526^), to more accurately investigate the binding features in correspondence with the N-terminal aromatic residue Tyr498 and Phe501, the simulations were extended by also considering the adjacent region L^490^LASPRSC^497^, which is part of the PADI4 sequence but not of the peptide used in our experiments. As a consequence, the binding of the 16 fragments L^490^LASPRS^496^, A^492^SPRSCY^498^,…, K^520^KKQQKI^526^ was simulated. Therefore, our simulations are expected to reproduce more closely the binding to Impα3 of the predicted NLS2-PADI4 region of the native protein, rather than the binding of the isolated peptide in solution.

### 2.10. Western Blot

Different dilutions (40, 20, 10, and 5 µM) of PADI4 were mixed with 5 µL of NuPAGE^®^ (Invitrogen, Barcelona, Spain). They were separated by SDS-PAGE using 10% gels and transferred to a nitrocellulose membrane (Bio-Rad Laboratories Inc, CA, USA). Separated membranes were incubated with 1 µM Impα3, for 2 days, and then the membranes were washed three times for periods of 7 min with 1× phosphate buffer solution (PBS), 0.1% Tween-20 buffer. Next, the membranes were blocked for 1 h with 5% (*w*/*v*) milk in 1× PBS, 0.1% Tween-20 buffer. Finally, they were incubated overnight at 4 °C with primary antibody anti-KPNA4 (rabbit, 1:800, Quimigen, Madrid, Spain), followed by 1 h incubation at room temperature with ECL TM anti-rabbit IgG, horseradish peroxidase linker (GE Healthcare, Chalfont St Giles, UK). The membranes were visualized with ECL TM Prime Western blotting detection reagent (Amersham TM) in a ChemiDoc Bio-Rad instrument.

### 2.11. Size Exclusion Chromatography (SEC)

Size exclusion chromatography experiments were carried out as described [48] on an AKTA FPLC using a calibrated analytical Superdex 75 10/30 HR FPLC column (GE Healthcare, Barcelona, Spain) with both peptides in the following concentration ranges: 50–400 µM of protomer concentration for NLS1-PADI4 and 200–400 µM for NLS2-PADI4 peptide. The elution volumes were obtained from analyses with UNICORN software (GE Healthcare, Barcelona, Spain) from three different measurements. The void volume (7.54 ± 0.06 mL) was determined from blue dextran, and the bed volume (18.98 ± 0.03 mL) was determined from conductivity measurements in a Tris elution buffer (20 mM, pH 7.6, and 250 mM NaCl). Samples were eluted at a rate of 1 mL/min and continuously monitored with an online detector at a wavelength of 280 nm. Analyses were carried out as described [48]. The column was calibrated with the standard set of low-molecular-weight (GE Healthcare, Barcelona, Spain) globular proteins; as a comparison, ribonuclease A, with a molecular weight of 13.7 kDa, eluted at 13.3 mL in such a column.

## 3. Results

### 3.1. PADI4 Was Bound to Impα3 and ΔImpα3

To test whether PADI4 interacted with Impα3 and ΔImpα3 (i.e., the Impα3 truncated species lacking the IBB domain) in vitro, we followed a two-part experimental approach. Firstly, we used steady-state fluorescence and CD as spectroscopic techniques to observe a possible binding and concomitant conformational changes in the macromolecules; secondly, we used fluorescence and ITC to quantitatively measure the thermodynamic parameters of such binding.

**Figure 1:**
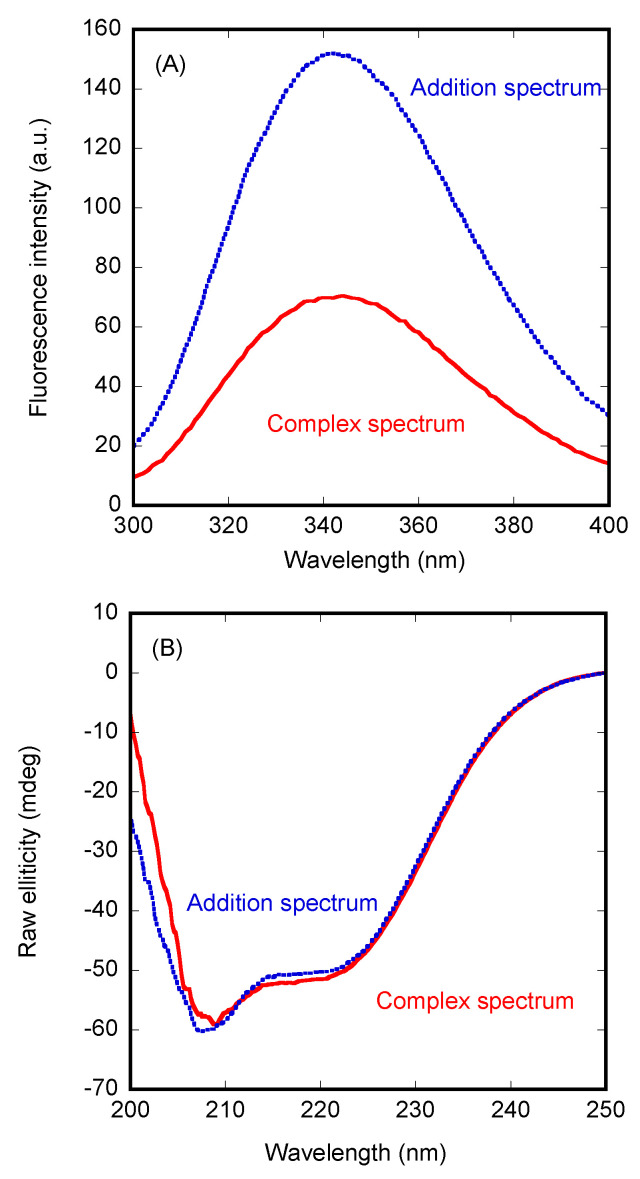
Binding of Impα3 to PADI4 as monitored by spectroscopic probes. (**A**) The spectrum of PADI4/Impα3 complex after excitation at 280 nm, and the addition spectrum obtained by the sum of those of the two separated macromolecules. (**B**) Far-UV CD spectrum of the PADI4/Impα3 complex, and the addition spectrum. All experiments were performed at 25 °C.

We used fluorescence to determine whether there was a change in (i) the position of the maximum wavelength, (ii) in the fluorescence intensity at that wavelength, or (iii) in both, when the spectrum of the complex was compared to that obtained from the addition of the separated spectra of the two isolated proteins. In the presence of Impα3, we observed a variation in the fluorescence intensity (after excitation at 280 nm) (Figure 1A), but there were no changes in the maximum wavelength of the spectrum. After excitation at 295 nm, similar variations were observed upon complex formation with Impα3. Furthermore, variations between the two spectra (i.e., the addition spectrum and that of the complex) were observed by excitation at both wavelengths (280 and 295 nm) when using ΔImpα3 to form the complex with PADI4 (Figure S1A). However, the variations were smaller than those observed when monitoring the binding to Impα3.

Next, we carried out far-UV CD measurements, trying to confirm the fluorescence binding results. In contrast to the observations for Impα3 described above, the addition spectrum was not very different from that of the complex (Figure 1B). A similar behavior was observed for ΔImpα3 (data not shown). Therefore, we can conclude that there were no large changes in the secondary structures of PADI4 or in those of the importin species when the two proteins were bound.

**Figure 2:**
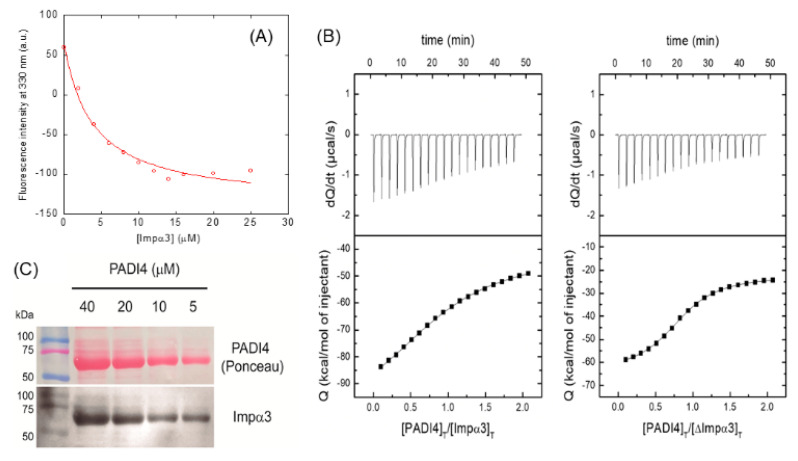
Binding of Impα3 to PADI4 as monitored by biophysical probes and Western blot analyses. (**A**) Titration curve monitoring the changes in the fluorescence at 330 nm when Impα3 was added to PADI4. The fluorescence intensity on the *y*-axis is the relative signal after removal of the corresponding blank. The line through the data is fitted to Equation (1). Experiments were carried out at 25 °C. (**B**) Calorimetric titrations for the PADI4 binding to (**left**) Impα3 and (**right**) ΔImpα3. Upper panels show the thermograms (thermal power as a function of time), and lower panels show the binding isotherms (ligand-normalized heat effects per injection as a function of the molar ratio in the calorimetric cell). Continuous lines correspond to the fitting curves according to a single ligand binding site interaction model. Experiments were carried out at 25 °C. (**C**) Polyacrylamide gels were loaded with several concentrations of PADI4 as visualized with Ponceau staining. Nitrocellulose membranes were later incubated with Impα3 to see the binding, washed, and revealed with the corresponding antibody against Impα3.

Since we observed changes in the fluorescence spectrum upon binding of PADI4 to Impα3 or ΔImpα3, we carried out titrations by keeping constant the concentration of PADI4 and increasing the concentration of the importin species. The results indicate (Figure 2A) that, for Impα3, the *K_d_* was 3.9 ± 0.8 µM, whereas, for ΔImpα3 (Figure S1B), the *K_d_* was 6 ± 1 µM, which are values quite comparable, within the fitting error.

We also used ITC to determine the thermodynamic binding parameters to both importin species (Figure 2B, Table 1). The result indicated that the interaction of both importins with PADI4 was highly exothermic (favorable enthalpic contribution and unfavorable entropic contribution to the Gibbs energy of binding). For Impα3, the *K_d_* was 4.8 ± 0.9 µM (which is similar to that obtained by fluorescence; see above), whereas, for ΔImpα3, the *K_d_* was 1.3.

To further confirm the binding of intact Impα3 to PADI4, we performed a series of in vitro experiments, using WBs, to detect protein–protein interactions with the recombinant, purified proteins. We used PADI4 as the prey protein and loaded at several concentrations (40 to 5 µM) in a gel. The proteins were transferred to a nitrocellulose membrane and incubated for 2 days with Impα3 (KPNA4) as bait protein. Subsequently, we washed and revealed the membrane with an antibody against Impα3. The results showed a decreasing signal of Impα3 binding to PADI4 according to the size and amount of loaded protein (Figure 2C). That is, the bait protein (Impα3) was detected on spots in the membrane where the prey protein (PADI4) was located, confirming that the two proteins formed a complex.

To sum up, we conclude that PADI4 could bind to each of the two importin species, with similar affinity constants. Moreover, our experiments confirmed the direct in vitro binding of Impα3–PADI4; however, we cannot rule out that a more complex interaction may be taking place in vivo where other partners could also be involved.

### 3.2. Conformational Features of the Isolated NLSs of PADI4

Since there was binding between PADI4 and both importin species, we wondered whether its two isolated NLS regions, predicted using the webserver cNLS Mapper, were also capable of binding to both importins. Earlier X-ray studies [47] and the results of other predictors of NLS sites (such as PSORT II, available at http://psort.hgc.jp/form2.html, accessed on 8 December 2021) only identified the first region, NLS1, whose sequence is P^56^PAKKKSTGSSTWPLDPGVEVTLTMKVASGS^86^; this region was also predicted to be an NLS using our reference NLS predictor, cNLS Mapper. However, the latter webserver also predicted another region, NLS2, corresponding to the sequence R^495^SCYKLFQEQQNEGHGEALLFEGIKKKKQQKI^526^ (with a score of 6.1, compared to a score of 5.7 for NLS1). Therefore, we had two distinct NLS predictions, and we decided to test the ability of the two corresponding isolated peptides, NLS1/2-PADI4 (see Section 2.3. for their sequences), to bind both importin species in solution. Before testing such ability, we carried out a biophysical and structural characterization of the isolated peptides in solution.

#### 3.2.1. Isolated NLS1-PADI4 Was Oligomeric and Disordered in Solution

**Figure 3:**
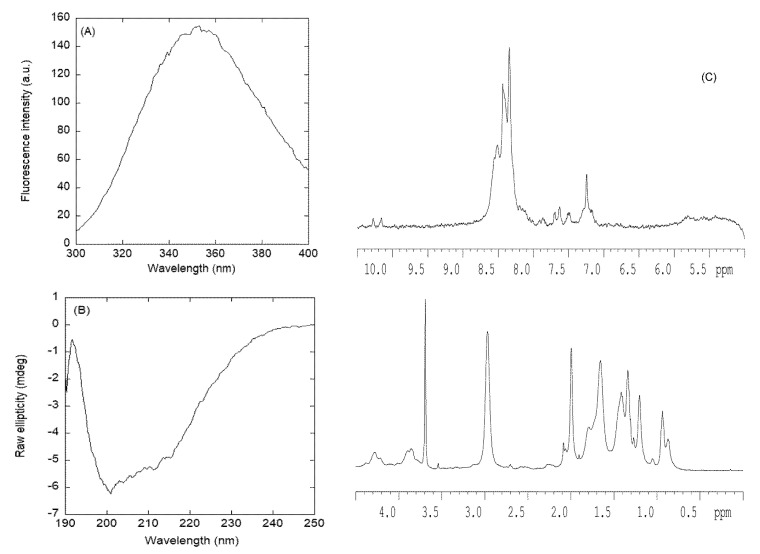
Conformational features of isolated NLS1-PADI4 in solution. (**A**) Fluorescence spectrum of NLS1-PADI4 in 20 mM Tris buffer (pH 7.5), 5 mM TCEP, 150 mM NaCl, and 5% glycerol at 25 °C. (**B**) Far-UV CD spectrum of NLS1-PADI4 at 25 °C in sodium phosphate buffer (50 mM, pH 7.5). (**C**) 1D-^1^H-NMR spectrum of isolated NLS1-PADI4 at 10 °C and pH 7.0 (100 mM, sodium phosphate buffer).

The fluorescence spectrum of NLS1-PADI4 had a maximum at ~350 nm due to the emission of its sole tryptophan, Trp68 (Figure 3A). The far-UV CD spectrum of isolated NLS1-PADI4 showed an intense band between 202 and 215 nm (Figure 3B), indicating that the peptide did not possess only a random-coil conformation. We deconvolved the CD spectrum using the k2D software on the DICHROWEB website [69,70,71]; the deconvolution yielded 9% α-helix, 37% β-sheet, and 54% random coil. The deconvolution using Contin or Selcon3 yielded similar results, with percentages of α-helix between 10% and 13%, of β-sheets between 20% and 24%, of β-turns between 15% and 21%, and of random coils between 45% and 52%. Therefore, all the predictors indicate that NLS1-PADI4 was mainly disordered, but with a relevant fraction of β-sheets. It could be suspected that, because of the presence of two proline residues in the central region of the polypeptide chain (Pro69 and Pro72), the peptide might also adopt a fraction of poly-proline II conformation; however, the far-UV CD spectrum (Figure 3B) lacked the positive band around 225 nm, which is a feature of this type of conformation [72]. The disordered character of NLS1-PADI4 was further confirmed by the 1D-^1^H-NMR spectrum (Figure 3C), with all the amide protons between 8.0 and 8.6 ppm, whereas the alkyl protons were clustered between 0.8 and 1.0 ppm. Furthermore, the indole proton of Trp68 appeared at 10.2 ppm, along with two signals due to the presence of a slow *cis*–*trans* isomerization equilibrium of the following residue Pro69 (Figure 3C). For those kinds of protons, all such values are typical of disordered polypeptide chains [59].

We also determined the hydrodynamic radius of the peptide in solution. First, the fitting of the measurement of the intensity of the methyl groups to a single exponential from the DOSY yielded a value of *D* and an estimated *R*_h_, obtained from the comparison with the *D* of dioxane (6.8 ± 0.3 × 10^−6^ cm^2^·s^−1^), of 8.8 ± 0.8 × 10^−7^ cm^2^·s^−1^ and 17 ± 2 Å, respectively. This value of *R*_h_ was slightly larger than that theoretically expected for a random-coil polypeptide [73] with such a molecular weight (2819.27 Da), i.e., 14.3 Å. This result suggests the presence of oligomeric species in solution. We could further confirm this hypothesis on the basis of two pieces of evidence. First, two-exponential fitting of the decay of the methyl intensity yielded a *D* of 4 ± 1 × 10^−7^ cm^2^·s^−1^, corresponding to an estimated *R*_h_ of 34 ± 6 Å, thus indicating the presence of a self-associated species; the second exponential led to the same *D* value, previously described with the fitting to a single exponential. According to the same expression used to calculate the *R*_h_ (R_h_ = 0.027 MW^1/2^, where *R*_h_ is assumed to be in nm and MW represents the mass in Da [73]), we can estimate the molecular weight for those species in solution as 16,421.9 Da, which suggests the presence of a hexameric species (considering an MW of 2819.27 Da for the monomer). Second, attempts to obtain a good TOCSY spectrum (by varying the length of the different mixing, spin-lock sequences used) to assign the resonances of NLS1-PADI4 failed; this result is indicative of polypeptide chains with a short relaxation time, such as those associated with an oligomer with a large molecular weight [59]. Chromatograms of a solution containing NLS1-PADI4 resulted in a peak with strong tailing centered at 14.82 mL (Figure S2). This resulted in a Stokes radius, according to the weight-average partition coefficients relationships [48], of 10.7 Å. The strong tailing is indicative of equilibria among several species with different molecular weights.

To conclude, the canonical NLS1-PADI4 species was disordered and had a tendency to self-associate at physiological pH.

#### 3.2.2. Isolated NLS2-PADI4 Was Monomeric and Disordered in Solution

**Figure 4:**
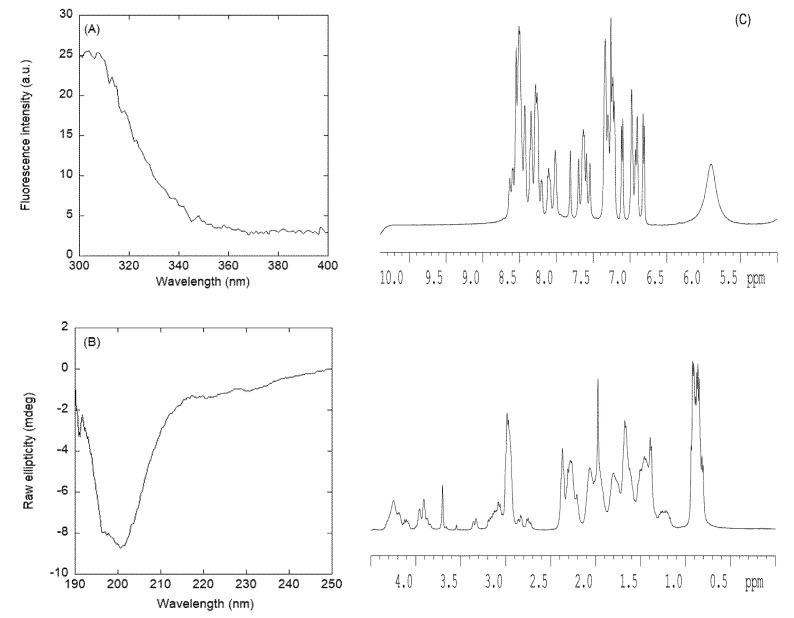
Conformational features of isolated NLS2-PADI4 in solution. (**A**) Fluorescence spectrum of NLS2-PADI4 in 20 mM Tris buffer (pH 7.5), 5 mM TCEP, 150 mM NaCl, and 5% glycerol at 25 °C. (**B**) Far-UV CD spectrum of NLS2-PADI4 at 25 °C in sodium phosphate buffer (50 mM, pH 7.5). (**C**) 1D-^1^H-NMR spectrum of isolated NLS2-PADI4 at 10 °C and pH 7.0 (100 mM, sodium phosphate buffer).

The fluorescence spectrum of NLS2-PADI4 had a maximum at 308 nm due to the emission of its sole tyrosine, Tyr498 (Figure 4A). The far-UV CD spectrum of isolated NLS2-PADI4 showed an intense minimum at ~202 nm (Figure 4B), indicating that the peptide possessed mostly a random-coil conformation; the spectrum was completely different from that obtained for NLS1-PADI4, although we cannot rule out that the absorbance of the sole Trp68 in the NLS1-PADI4 spectrum in the interval 210–220 nm [74,75,76] could alter its shape. We tried to deconvolve the far-UV CD spectrum of NLS2-PADI4 by using the k2D software on the DICHROWEB website [69,70,71]; the deconvolution yielded a fraction of 5% α-helix, 40% β-sheet, and 55% random coil. Deconvolution results obtained using Contin and Selcon3 yielded fractions of 7% to 9% for α-helices, 15% to 18% for β-sheets, 7% to 13% for β-turns, and 64% for random coils. Therefore, the percentages of the structure obtained in the deconvolution of far-UV CD spectrum for NLS2-PADI4 were similar to those obtained for NLS1-PADI4 (see Section 3.2.1), and NLS2-PADI4 was mainly disordered, with a high percentage of transient β-sheets. The disordered characteristic of NLS2-PADI4 was further confirmed by the 1D-^1^H-NMR spectrum (Figure 4C), with all the amide protons between 8.0 and 8.6 ppm, whereas the methyl protons were clustered between 0.8 and 1.0 ppm. In both cases, these values are observed in disordered chains [59].

On the other hand, the peptide was monomeric, as concluded from the value of *D* measured by the DOSY experiment and the estimated *R*_h_ (obtained from the comparison with the *D* of dioxane): 9.6 ± 0.3 × 10^−7^ cm^2^·s^−1^ and 15.1 ± 0.8 Å, respectively. This value of *R*_h_ was similar to that obtained theoretically for a random-coil polypeptide [59] with a corresponding molecular weight (3501.98 Da), i.e., 15 ± 2 Å. The SEC experiments for NLS2-PADI4 yielded an elution peak at 15.15 mL (Figure S2). As the molecular weight of NLS1-PADI4 was slightly larger than that of NLS2-PADI4, but it eluted at larger elution volumes, the actual molecular weight of the NLS1-PADI4 species loaded in the column (even taking into account of the dilution effect of the bed volume) must correspond to an oligomeric species. The elution volume of NLS2-PADI4 resulted in a Stokes radius of 9.64 Å, lower than that of NLS1-PADI4 (see previous section).

To further confirm the mainly disordered nature of NLS2-PADI4, we also carried out homonuclear 2D-^1^H-NMR experiments (Table ST1); in this case, we were able to obtain good TOCSY spectra to allow us to assign the resonances. However, we could not fully assign them due to the large number of lysine, glutamine, and glutamic acid residues. NLS2-PADI4 was mainly disordered in solution, as suggested by different evidence, further confirming the results from far-UV CD (Figure 4B) and 1D-^1^H-NMR spectra (Figure 4C). Firstly, the conformational shifts (Δδ) of H_α_ protons [59,60,61] for those unambiguously assigned amino acids were within the commonly accepted range for random-coil peptides (Δδ ≤ 0.1 ppm) (Table ST1). Secondly, no long- or medium-range NOEs were observed in the spectra, but only consecutive ones (i.e., αN (*I*, *i*+1) and βN (*i*, *i*+1)) were observed in the polypeptide patches fully assigned.

Taken together, all the experimental techniques concurred that the isolated NLS2-PADI4 was monomeric and disordered in aqueous solution.

### 3.3. Isolated NLS1/2-PADI4 Could Bind to Each of the Importin Species

Next, we wondered whether the isolated NLS1/2-PADI4 peptides were capable of binding to both importins (Impα3 and ΔImpα3), and, if so, we wanted to measure their binding affinity for each of them. We followed the same procedure used for intact PADI4; that is, first we tried to detect binding by using fluorescence and far-UV CD, and then we tried to measure such binding quantitatively using fluorescence, ITC, and BLI.

**Figure 5:**
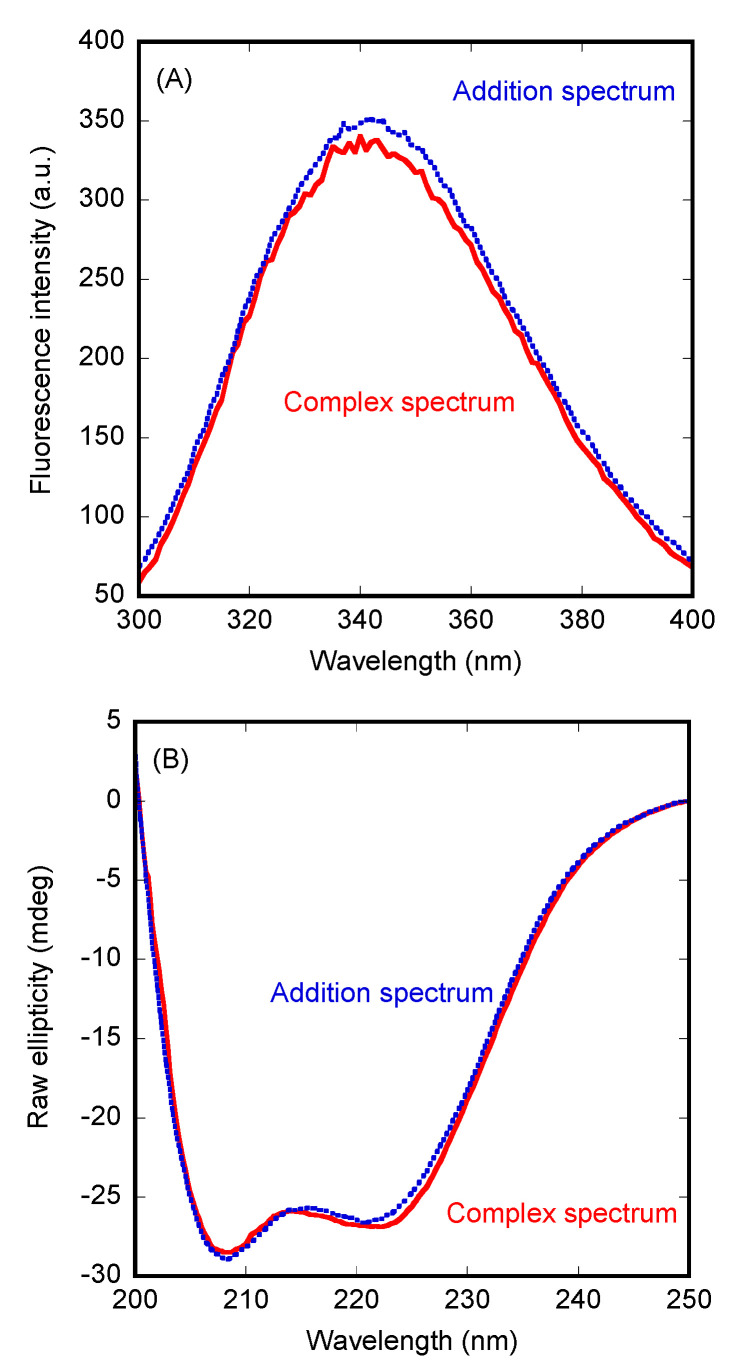
Binding of importin species to NLS2-PADI4 as monitored by spectroscopic probes. (**A**) Fluorescence spectrum of the complex ΔImpα3/NLS2-PADI4 and that obtained by the addition of the spectra of the two isolated molecules. (**B**) Far-UV CD spectrum of the complex Impα3/NLS2-PADI4 and that obtained by the addition of the spectra of the two isolated molecules. Experiments were carried out at 25 °C.

Fluorescence experiments showed that there were no large changes in the spectra upon addition of NLS2-PADI4 to Impα3/ΔImpα3, but there were large ones upon addition of NLS1-PADI4 to each of the importin species, probably because of the presence of Trp68 in the latter peptide (Figure 5A and Figure S3A). Furthermore, the far-UV CD spectrum of the complex and that obtained by the addition of the two isolated spectra showed no changes for NLS2-PADI4 (Figure 5B), but there were large changes for NLS1-PADI4 with both importins (Figure S3B). As the peptide had a smaller size than that of the two importins, these results suggest that the secondary conformational preferences of NLS1-PADI4 changed dramatically in the presence of any of the two importins. We could not rule out that the structure of the importins changed as well, although this is unlikely for a well-folded protein with an organized and repetitive ARM structure. Furthermore, the tertiary environment around one of the tryptophans of the importins and/or Trp68 from NLS1-PADI4 changed when both macromolecules were present in solution. On the other hand, the absence of changes in the far-UV CD spectra when NLS2-PADI4 was present in solution could mean that (i) neither NLS2-PADI4 nor importin species changed their secondary structures upon binding, or (ii) CD did not report any conformational change in the polypeptide chains (i.e., CD was spectroscopically silent). However, the lack of variations in the CD spectra did not rule out the possibility that the binding took place, as we used other biophysical probes to test it. In fact, we attempted to determine the binding affinity of both peptides for each importin species using (i) ITC, (ii) BLI, and (iii) the small changes observed in fluorescence intensity for NLS2-PADI4 and the large ones for NLS1-PADI4.

**Figure 6:**
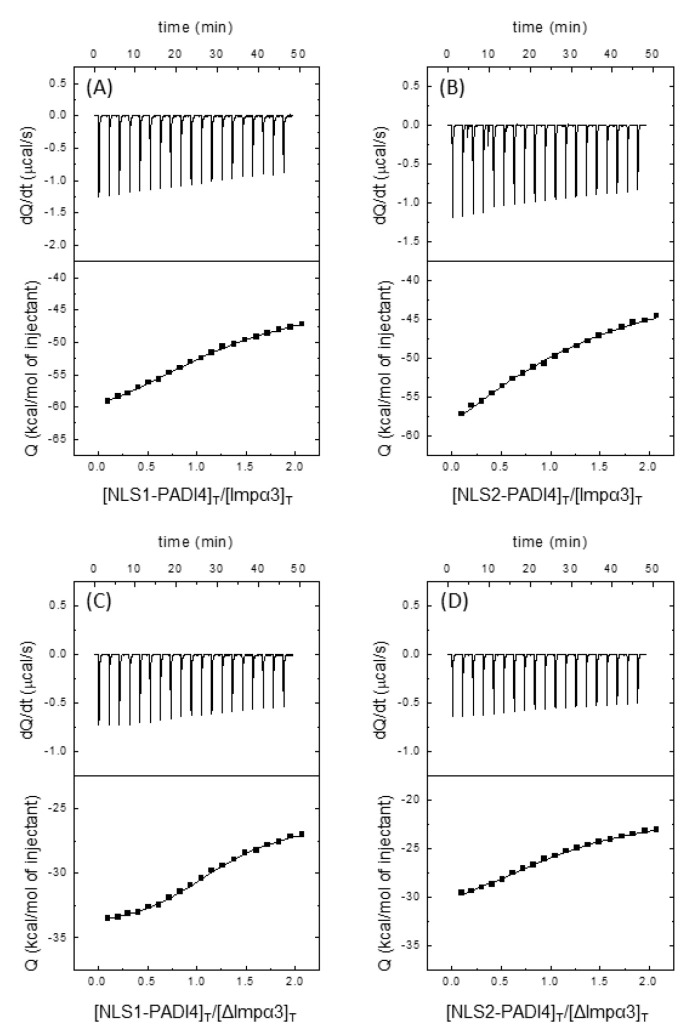
Binding of importin species to NLS1-PADI4 and NLS2-PADI4 as monitored by ITC. Calorimetric titrations for the NLS peptides binding to importin species. Impα3 interacting with (**A**) NLS1-PADI4 and (**B**) NLS2-PADI4, and ΔImpα3 interacting with (**C**) NLS1-PADI4 and (**D**) NLS2-PADI4. Upper panels show the thermograms (thermal power as a function of time), and lower panels show the binding isotherms (ligand-normalized heat effects per injection as a function of the molar ratio in the calorimetric cell). Continuous lines correspond to the fitting curves according to a single ligand binding site interaction model. Experiments were carried out at 25 °C.

The calorimetric titrations with both peptides revealed that both polypeptide chains were capable of binding to both importins, Impα3 and ΔImpα3, with a favorable enthalpic contribution and an unfavorable entropic contribution to the Gibbs energy of binding, with NLS2 showing a more exothermic binding (Figure 6, Table 1). In both cases, we observed a single binding reaction, in contrast to what was found for other NLS peptides, where two transitions were observed and explained as due to the simultaneous binding to the major and minor binding sites of importin [77]. For NLS1-PADI4, the *K_d_* values were 4.4 ± 4 µM for Impα3 and 1.5 ± 1 µM for ΔImpα3; for NLS2-PADI4, the *K_d_* values were 23 ± 10 µM for Impα3 and 4.3 ± 4 µM for ΔImpα3. Thus, NLS1-PADI4 showed a slightly higher affinity, compared to NLS2-PADI4, for both importins, and ΔImpα3 showed a slightly higher affinity, compared to Impα3, for NLS1/2-PADI4.

**Figure 7:**
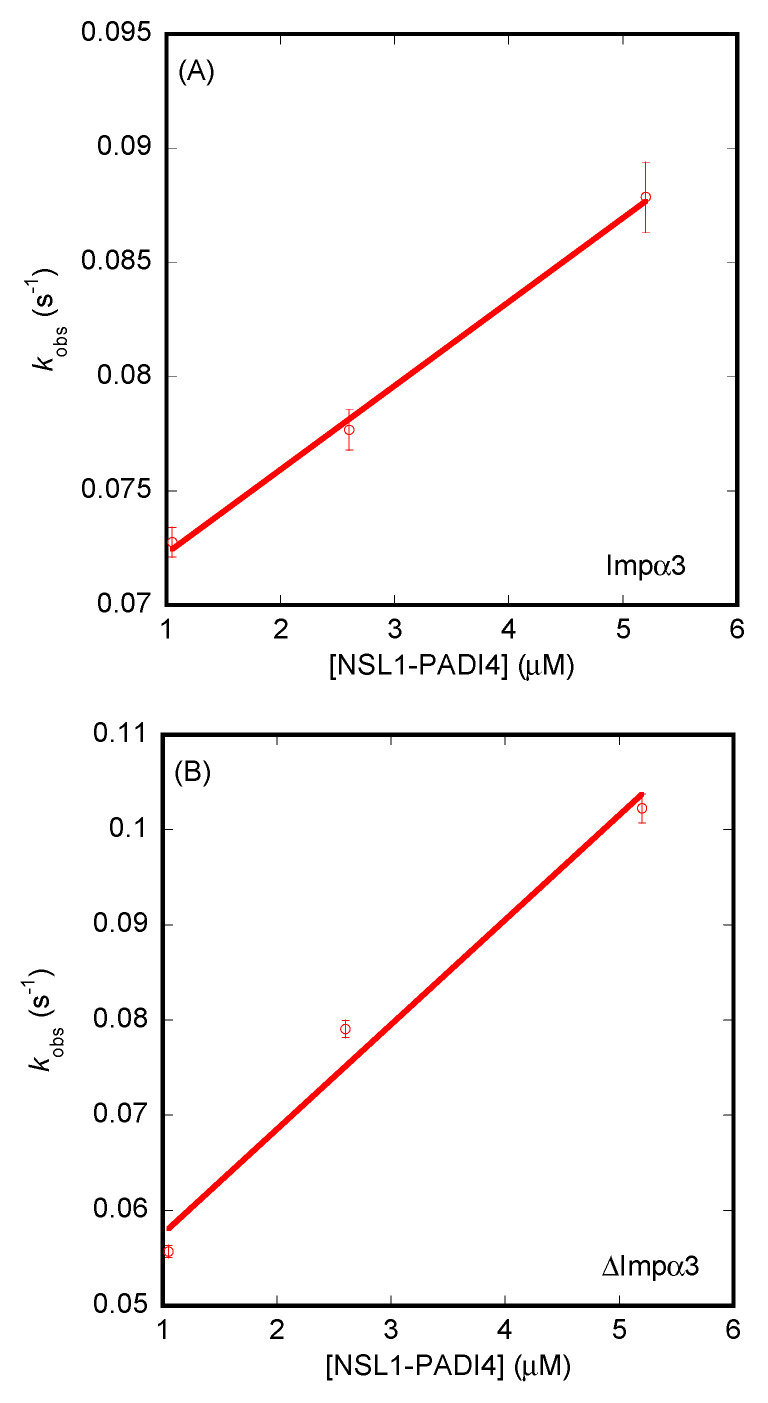
Binding of importin species to NLS1-PADI4 as monitored by BLI. (**A**) Pseudo-first-order plot of the binding of the peptide to Impα3 (Equation (6)). (**B**) Pseudo-first-order plot of the binding of the peptide to ΔImpα3 (Equation (6)). The error bars in both panels are fitting errors to the exponentials of the sensorgrams. Experiments were carried out at 25 °C.

**Figure 8:**
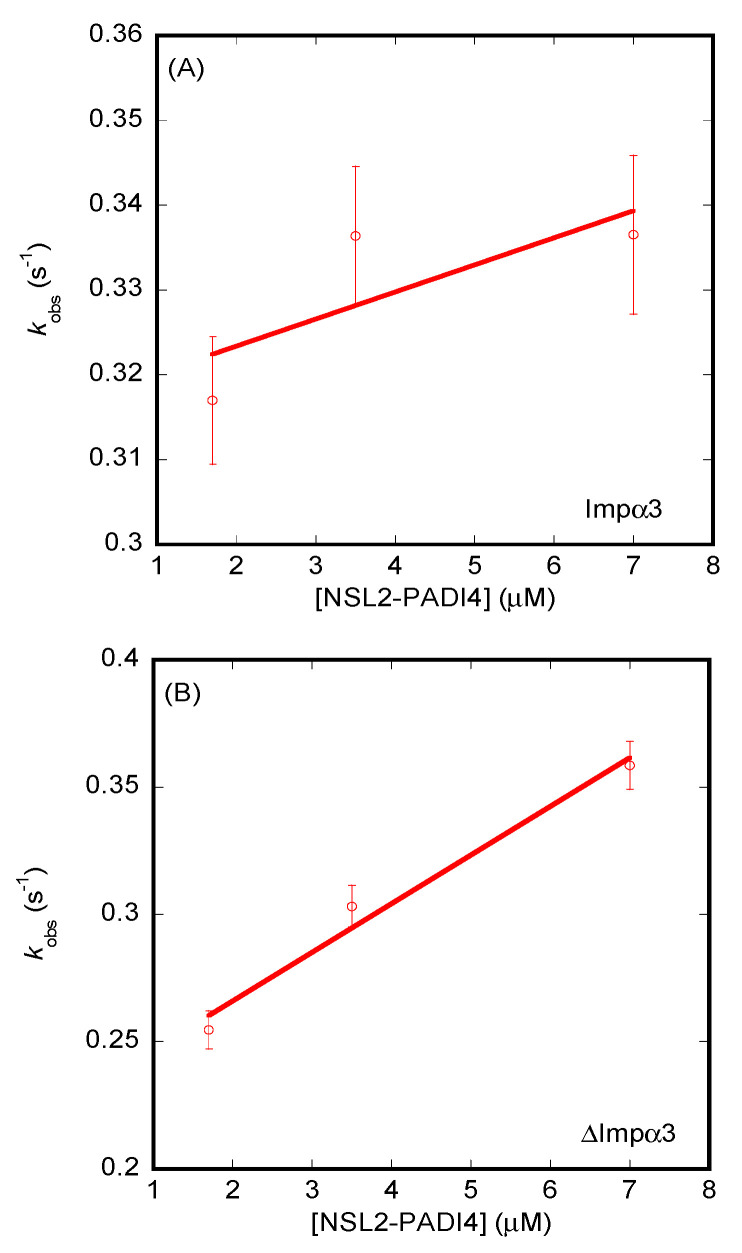
Binding of importin species to NLS2-PADI4 as monitored by BLI. (**A**) Pseudo-first-order plot of the binding of the peptide to Impα3 (Equation (6)). (**B**) Pseudo-first-order plot of the binding of the peptide to ΔImpα3 (Equation (6)). The error bars in both panels are fitting errors to the exponentials of the sensorgrams. Experiments were carried out at 25 °C.

The results from BLI (Figure S4) yielded values of the dissociation constants different from those measured for ITC. For NLS1-PADI4, the *K_d_* values were 18 ± 4 µM (for Impα3), with *k_on_* = 0.0037 ± 0.0002 µM^−1^·s^−1^ and *k_off_* = 0.0685 ± 0.0007 s^−1^ (Figure 7A), and 4 ± 1 µM (for ΔImpα3), with *k_on_* = 0.011 ± 0.002 µM^−1^·s^−1^ and *k_off_* = 0.046 ± 0.005 s^−1^ (Figure 7B). On the other hand, for NLS2-PADI4, the values of the dissociation constants and kinetic rates were 99 ± 10 µM (for Impα3), with *k_on_* = 0.003 ± 0.002 µM^−1^·s^−1^ and *k_off_* = 0.32 ± 0.01 s^−1^ (Figure 8A), and 12 ± 4 µM (for ΔImpα3), with *k_on_* = 0.020 ± 0.003 µM^−1^·s^−1^ and *k_off_* = 0.23 ± 0.01 s^−1^. Therefore, from the kinetic point of view, we can conclude that (i) both peptides had an affinity for ΔImpα3 higher than that for Impα3 (i.e., the dissociation equilibrium constant for ΔImpα3 was smaller than for Impα3), in agreement with the ITC experiments, (ii) NLS1-PADI4 showed always a higher affinity than NLS2-PADI4 for any of the importin species, in agreement with the ITC experiments, (iii) the values of the *k_on_* rates for both peptides were one order of magnitude lower for Impα3 than for ΔImpα3, indicating that the association of both peptides to the latter importin species was faster, and (iv) the values of the dissociation rates from both importin species, *k_off_*, were smaller (one order of magnitude) for NLS1-PADI4 than for NLS2-PADI4. Taken altogether, we can conclude that the binding reaction was modulated by the nature of the importin species (with or without the IBB) and each particular NLS region. Although the values for the dissociation constant determined by ITC and BLI might seem somewhat different, the relative differences (that is, the respective fold changes) for the two importin species and for the two NLS peptides were similar.

**Figure 9:**
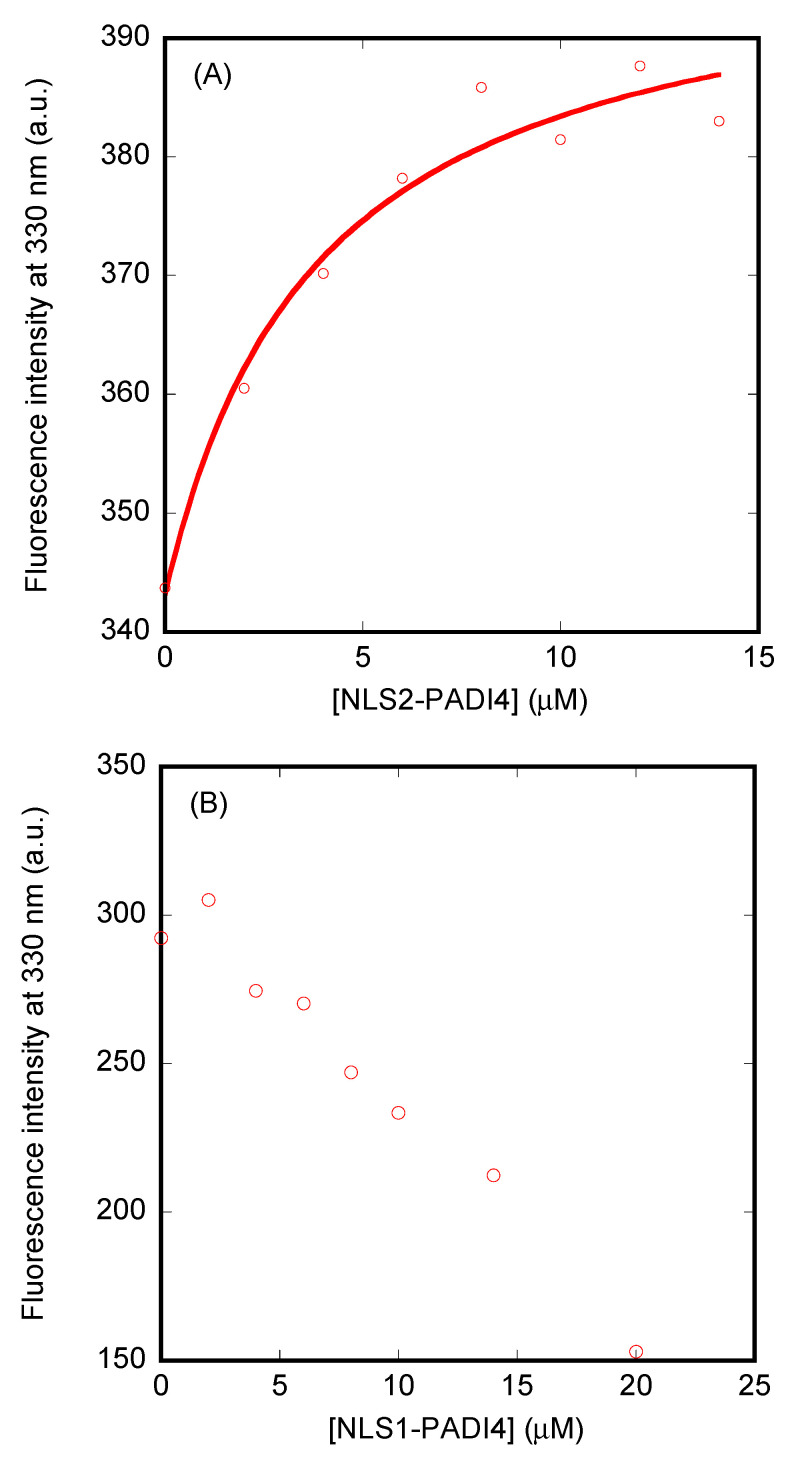
Binding of importin species to NLS1- and NLS2-PADI4 as monitored by fluorescence: (**A**) Titration curve monitoring the changes of fluorescence at 330 nm when NLS2-PADI4 was added to ΔImpα3. The fluorescence intensity on the *y*-axis is the relative fluorescence intensity after removal of the corresponding blank. The line through the data is fitted to Equation (2). (**B**) Titration curve monitoring the changes in the fluorescence at 330 nm when NLS1-PADI4 was added to Impα3. The fluorescence intensity on the *y*-axis is the relative fluorescence intensity after removal of the corresponding blank. All experiments were carried out at 25 °C.

The changes observed by fluorescence, even though they were small (see above), provided values of the dissociation constants very similar for both importin species when bound to NLS2-PADI, i.e., 4 ± 2 µM (for Impα3) and 4 ± 1 µM (for ΔImpα3) (Figure 9A). These values were smaller than those obtained by BLI for the same peptides, indicating that the binding process probably followed a non-two-state mechanism. Conversely, although the fluorescence changes for NLS1-PADI4 were far larger than for NLS2-PADI4, we could not monitor a reasonable titration curve within the constraints given by the experimental error (Figure 9B).

### 3.4. NLS1/2-PADI4 Could Bind to the Major NLS Binding Site of Impα3

The binding of the two predicted NLS regions of PADI4 to Impα3 was screened in detail using molecular docking. This simulation technique does not take into account the protein dynamics and, therefore, is not sensitive to the presence or absence of the IBB domain. For this reason, the importin was modeled in the sole IBB-depleted form (ΔImpα3), and results were assumed to be approximately valid for both importin species. Furthermore, molecular docking cannot accurately treat the binding of the whole NLS1-PADI4 or NLS2-PADI4 peptide to Impα3, because the peptides possess too many degrees of freedom (105 and 136 rotatable dihedral angles for NLS1-PADI4 and NLS2-PADI4, respectively). Nevertheless, both the major and the minor NLS-binding sites of Impα3 are too small to accommodate all the residues included in the two peptides (27 and 29 residues for NLS1-PADI4 and NLS2-PADI4, respectively); therefore, the core binding region of both peptides is expected to be much shorter. As an example, in the case of the crystallographic complex between Impα3 and the NLS sequence of the Ran-binding protein 3 [65], the anchoring sequence constituting the binding interface was significantly shorter. For this reason, according to a screening protocol we already successfully employed in other cases [78,79,80,81], the docking was performed by considering seven-residue fragments of the two peptides. Each of these fragments differed by a shift of two consecutive residues with respect to the following one, and the whole set of fragments covered the whole sequence of the two NLS peptides.

**Figure 10:**
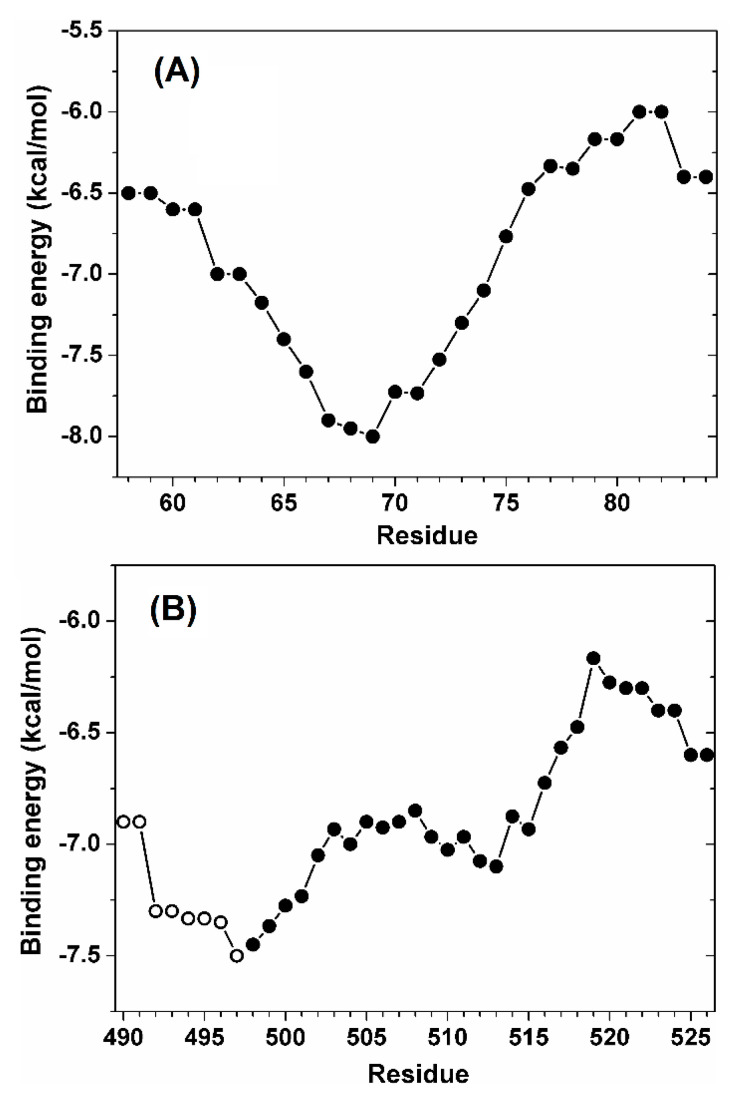
Affinity of the two NLS regions of PADI4 toward Impα3 estimated in molecular docking simulations. (**A**) Peptide NLS1-PADI4, encompassing residues 58–84. (**B**) The second NLS2 region of PADI4, including the N-terminal residues 490–497 (open symbols) and the peptide NLS2-PADI4 encompassing residues 498–526 (solid symbols). The affinity was calculated for seven-residue fragments, and values reported for each residue are the average over all simulation runs including that residue.

The binding affinities calculated for the two peptides are shown in Figure 10, with the energy value for each peptide residue corresponding to the average of the binding scores of all fragments that contained that residue. The binding score for the peptide NLS1-PADI4 (Figure 10A) had a minimum in the curve at about −8.0 kcal/mol, and the affinity of this region was more favorable by >1 kcal/mol compared to the other regions of the same NLS1-PADI4. This energy value is slightly more favorable compared to the range (from −7.9 to −7.2 kcal/mol) we found in analogous docking experiments between Impα3 and nine- or eight-residue-long fragments of the NLSs of two intrinsically disordered proteins, NUPR1 and its paralog NUPR1L, for which the binding was also experimentally confirmed [41,42]. Thus, the docking simulations suggested a favorable binding affinity in the low micromolar range. The core binding region of the NLS1-PADI4 included the key protein residue Trp68 and encompassed residues 66–72, confirming our initial assumption that a restricted seven-residue region drives the binding of the whole 27-residue peptide.

For NLS2-PADI4, the curve did not show a clear minimum in the binding energy when the sole peptide was considered (Figure 10B, solid symbols). This observation suggested the necessity to extend the exploration beyond the N-terminal region of this peptide. It is also important to note that the estimation of the binding score is less accurate for residues belonging to the two main chain termini, because they were obtained from averages of a lower number of docking experiments compared to the values obtained for amino acids far away from the termini. As an example, the binding contribution of the aromatic residue Tyr498 in this region, in the absence of other information, could only be assumed as equal to the docking score calculated for the fragment Y^498^KLFQEQ^504^, if the sole NLS2-PADI4 sequence were to be considered. For this reason, a longer portion of the sequence of PADI4 including eight more residues (490–497) was explored in our simulations (Figure 10B, open symbols). Thus, the results are representative of the binding of a larger region of PADI4 sequence (residues 490–526), more than representing the sole NLS2-PADI4 peptide (residues 498–526). The overall curve showed an energy minimum at about −7.5 kcal/mol, a value only slightly less favorable compared to the one obtained for NLS1-PADI4, predicting that the two NLS regions of PADI4 should bind Impα3 with a similar affinity. The core binding region in the case of NLS2-PADI4 encompassed the amino acids 495–501 and confirmed the involvement of the key residue Tyr498 and a few other adjacent amino acids, all belonging to the seven-residue fragment located at the N-terminal region of the NLS2-PADI4 peptide.

**Figure 11:**
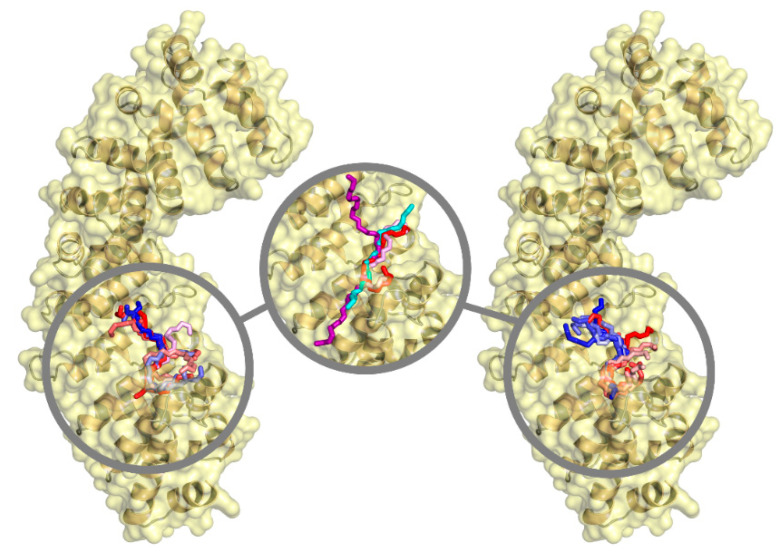
Binding location of seven residue long fragments of the two NLS regions of PADI4 on the surface of Impα3 observed in simulations. (**Left**) conformations of peptide NLS1, and (**right**) peptide NLS2 region. Fragments follow the color scheme red → magenta → blue, going from the N to the C terminus of the two NLS peptides. (Center) comparison among the fragments with the most favorable binding affinity of (magenta) NLS1-PADI4 and (red) NLS2-PADI4, and (cyan) crystallographic pose [66] of the NLS of the Ran-binding protein 3 (purple) and the Epstein–Barr virus EBNA-LP protein (cyan) bound to the major binding sites of Impα3 and Impα1, respectively. For clarity, the sole backbone chain is shown for all the sequences.

The analysis of the docking results was also extended to detail the binding location of both NLS regions of PADI4 on the surface of Impα3. The best binding modes of all the seven-residue fragments of the two peptides are reported in Figure 11. These docking poses were all found in correspondence with the ARM repeats 2–4 of Impα3, which correspond to the major protein binding site for NLS of cargo proteins [82,83]. The fact that only the major binding site of Impα3 was involved is further supported by the fact that a single binding reaction was observed in the ITC experiments (Figure 6), conversely to what was observed for other peptides encompassing classical bipartite NLSs [77]. A moderate affinity of a larger variety of adjacent fragments toward the same target site could be useful in the first steps of the binding recognition process, although the sole two fragments expected to anchor in that position at the end of the association process are the core binding regions of PADI4 previously detected (residues 66–72 and 495–501), due to their more favorable binding affinity compared to the other fragments. A further comparison with the NLS of the Ran-binding protein 3, whose conformation in the complex with Impα3 is known at atomic detail in crystallography [65], shows that the two core binding regions of the NLS peptides of PADI4 are capable of anchoring in a similar way in the same location (as shown in Figure 11), with cation–π interactions between the aromatic residues from the corresponding NLS and positively charged residues from Impα3. Similar types of interactions have been observed in some atypical NLS when bound to importin at the minor binding site [84]. The binding interface of Impα3 also encompassed a restricted number of residues, with Trp184 and Trp231 forming van der Waals interactions that maintain an anchoring with PADI4.

We also report that similar results were obtained using, as a template to build Impα3, the structure extracted from the complex between Impα1 and the NLS of the EBNA-LP protein [66], which is the reference structure we previously used in other studies [41,42,43]. The predicted affinity curves (Figure S5) had, in general, a different shape compared to those found using Impα3 extracted from the complex with the Ran-binding protein 3; nevertheless, the binding energy at the minima were similar (variations were ≤0.5 kcal/mol), and the position of such minima differed by at most three residues along the NLS sequence of PADI4. Furthermore, even in this case, all the poses (Figure S6) were found in correspondence with the major binding site for cargo proteins, and they overlapped with the crystallographic position of the NLS. It is also evident (Figure 11 and Figure S6) that the core region of both crystallographic NLSs considered, as well as the two most favorable binders among the seven-residue-long fragments of the NLSs of PADI4, essentially overlapped.

## 4. Discussion

PADI4 is a key enzyme for the conversion of arginine to citrulline, an important PTM, and the only member of PADI family observed in both the nucleus and the cytoplasm under ordinary physiological conditions. Thus, the nuclear translocation of this protein is of special interest in cancer, and this work represents a first step to investigate this process. Our results revealed that PADI4 was capable of binding to Impα3, a nuclear carrier of the importin family, and its IBB domain-depleted species, ΔImpα3. Moreover, we showed that the use of the NLS predictor cNLS Mapper returned two potential NLS regions in PADI4, approximately located at the two termini of its polypeptide chain: (i) from Pro56 to Ser83, and (ii) from Arg495 to Ile526. Whereas the first region was already suspected to be an NLS of PADI4 using earlier NLS predictors and on the basis of the crystallographic structure of the protein [47], the latter constitutes a novelty. Both regions are solvent-exposed in the folded structure of PADI4 and, therefore, available to bind Impα3 with no impediment (Figure S7). Thus, we characterized the conformational propensities of the peptides corresponding to the isolated fragments of the two regions, and we measured their binding affinity for a specific importin, Impα3, for which we had already measured the affinity toward other cargos [41,42,43]. According to our in vitro measurements, both isolated regions were responsible for PADI4 binding to Impα3, as further confirmed by the molecular docking results (Figure 10 and Figure 11). The first region, from Pro56 to Ser83 (NLS1-PADI4), could not be classified as a classical monopartite NLS [28], as the core binding region comprises residues Thr67–Gly73. It has been suggested [22], on the basis of transfected cells and deletion mutants of PADI4 containing the region 45–74, that the Lys59–Lys60–Lys61 region might be important for nuclear translocation of PADI4. Our simulation studies indicate that, although such a region is involved in binding to Impα3, the core polypeptide patch is around the two central prolines flanked in the sequence by a tryptophan residue. Other examples of NLS regions that involve aromatic residues in their sequences have also been previously reported in the literature [28]. The second region, Arg495 to Ile526 (NLS2-PADI4), could not either be classified as a classical monopartite sequence, encompassing the core region of Tyr498–Gln504. As it happens with other proteins that are importin-dependent for their nuclear translocation, tryptophan residues of Impα3 and ΔImpα3 seemed to intervene in the binding, as we were able to follow the fluorescence titrations at 280 or 295 nm (Figure 9), and these findings were validated by our molecular docking calculations (Figure 10).

The main conclusions from our simulations can be summarized as follows: firstly, the whole sequence of both investigated NLS sequences of PADI4 has a distinct preference to interact with Impα3 in correspondence with the major NLS-binding site of this karyopherin. Secondly, in both cases, the core binding region of the NLS sequences corresponds to short fragments of about seven residues, corresponding to the central region of NLS1-PADI4 (residues 66–72) and the N-terminal region of NLS2-PADI4 (residues 495–501). Thirdly, in both cases, these core binding regions of the NLS peptides are located in correspondence with aromatic residues of PADI4, i.e., Trp68 and Tyr498 for, respectively, NLS1-PADI4 and NLS2-PADI4, acquiring a disordered conformation when bound. Fourthly, the binding patch on the carrier Impα3 is similarly small, albeit consisting of a surface region on the tertiary structure of the protein that is not restricted to a single portion of the protein sequence. Fifthly, the binding residues of Impα3 also include two aromatic amino acids, i.e., Trp184 and Trp231. Lastly, the binding energy between the two protein interfaces that drives the formation of the complex is between −7.5 and −8.0 kcal/mol, comparable to the values found in both simulations and experiments for the binding of other NLS polypeptides to Impα3 [41,42], and consistent with our in vitro experiments with NLS1/2-PADI4. In fact, according to ITC experiments, the binding of NLS1-PADI4 and NLS2-PADI4 to ΔImpα3 was characterized by Gibbs energy changes of −7.9 kcal/mol, and −7.3 kcal/mol, respectively. Moreover, it is not unusual that atypical and poorly basic NLSs, as those described here for PADI4, have aromatic residues involved in binding to importin (see, for instance, [28] and references therein).

We also demonstrated that both isolated NLS1/2-PADI4 peptides were disordered, and that at least NLS2-PADI4 did not have any propensity to acquire helix- or turn-like conformations, although we could not rule out the presence of local kinks around the two prolines in the sequence of NLS1-PADI4 (Pro69 and Pro72). The predicted hotspot of the importins for the association of both NLS1/2-PADI4 peptides is the major binding site for the NLSs of cargo proteins (Figure 11), as shown by the in silico experiments. Thus, we can conclude that NLS1/2-PADI4 behaved similarly to any other NLS regions belonging to a well-folded protein [29,40,85,86] or to IDPs [41,42,43].

Since PADI4 is a Ca(II)-dependent enzyme, it could be thought that the binding detected in this work might be affected by the presence of the ion. However, the presence of Ca(II) does not alter the monomer–dimer equilibrium of the enzyme [24,87]. Furthermore, the structural changes occurring in the presence of Ca(II) are mainly located at the C-terminus, where the active site is [47], and the two NLSs regions described here are far away from those regions where the changes in the presence of Ca(II) were detected (Ile313–Ile320, Pro338–Met348, Pro371–Pro387, Pro396–Gly403, Phe633–His644, and Ala351–Ala359) [47]. Lastly, the presence of Ca(II) was shown only to stabilize the presence of an unfolded intermediate either by using urea or temperature [24,88]. Therefore, the presence of Ca(II) can be safely assumed to not affect binding of PADI4 to Impα3 or its truncated species, although further studies will be necessary to dissect this point.

Previous studies with other NLSs of IDPs [41,42,43,82] or, alternatively, of folded proteins [29,40,83] suggest an inhibitory action of the IBB, which hampers binding of the NLS of the corresponding cargo protein into the major NLS-binding region of Impα3. Since ITC is considered the gold-standard for determining binding thermodynamic parameters, we focused on the dissociation constants measured by this technique. In this respect, we observed a slightly higher affinity of binding to NLS1/2-PADI4 for ΔImpα3 compared to Impα3, in agreement with the findings obtained for other NLS peptides assayed before [41,42,43]. The same conclusion about the values of the affinity constants of the two peptides for both importin species could be obtained from BLI experiments. Keeping in mind that the binding of the peptides might be a non-two-state process, we could observe that the apparent dissociation constants for Impα3 were always larger (i.e., lower affinity) than those for ΔImpα3. This difference between the two importin species may be due to the fact that the 60-residue-long IBB is competing with the NLS for the NLS-anchoring region. From a kinetic point of view, we observed that the binding of both peptides was faster to ΔImpα3, probably due to the absence of IBB and an associated kinetic barrier, as it is not necessary to displace that domain from the importin to allow access to the NLS binding site. As there are two NLSs in the same protein, we can speculate that either region can be used during nuclear translocation through NPC, allowing for a stronger cooperative binding. Alternatively, they can be used indistinctly, depending on the environmental conditions; hence, PADI4 can modulate its binding to the importin under different circumstances. Lastly, the fact that the dissociation constants were different for the two NLS suggests that the binding behavior of the cargo protein–Impα3 complex seems to be modulated by subtle details within the sequence of each particular NLS. These findings would open the avenue to selectively hamper nuclear translocation and might lead to the development of new molecular anticancer therapies.

## 5. Conclusions

PADI4 is found in both the nucleus and the cytoplasm, and it is present in several types of cancer tissues and various cells of the human innate immune system. This protein has two predicted NLS regions in its sequence, which can be recognized by importins for nuclear translocation. In this work, we demonstrated that PADI4 was capable of binding to both Impα3 and its IBB-depleted species, with a slightly higher affinity when the IBB domain was not present. As both PADI4 and importin appear to be overexpressed in some cancer types, and PADI4 must be transported within the nucleus to citrullinate histones and start de-condensation, the use of drugs hampering the binding of this enzyme to importin could provide a therapeutic approach to stop cancer progression. We also proved that the NLS regions of PADI4 were both capable of binding this nuclear carrier, and we suggested that a restricted core sequence provided an anchoring to the major NLS binding site for cargo proteins of Impα3. The results expand our knowledge on the molecular properties of PADI4, as well as support upcoming studies on the functional role of this protein.

## Figures and Tables

**Table 1:** Thermodynamic parameters of binding of PADI4 to the different macromolecules ^a^.

		*K_a_* (10^5^ M^−1^)	*K_d_* (μM)	Δ*H* (kcal/mol)	Δ*G* (kcal/mol)	-TΔS (kcal/mol)	n
PADI4	Impα3	2.1 (1.7, 2.5)	4.8 (4.0, 5.9)	−65.5 (−72.0, −60.5)	−7.3	−58.2	0.97 (0.94, 1.00)
	ΔImpα3	7.8 (7.3, 8.5)	1.3 (1.2, 1.4)	−38.4 (−39.5, 37.2)	−8.0	−30.4	0.84 (0.83, 0.85)
NLS1	Impα3	2.3 (1.6, 3.0)	4.3 (3.3, 6.3)	−21.3 (−25.3, −18.8)	−7.3	−14.0	1.24 (1.15, 1.37)
	ΔImpα3	6.5 (4.9, 8.3)	1.5 (1.2, 2.0)	−8.7 (−9.6, −8.0)	−7.9	−0.8	1.22 (1.16, 1.29)
NLS2	Impα3	0.43 (0.31, 0.57)	23 (18, 32)	−35.7 (−39.8, −31.1)	−6.3	−29.4	1.08 (0.98, 1.20)
	ΔImpα3	2.3 (1.5, 3.2)	4.3 (3.2, 6.7)	−11.2 (−13.7, −9.8)	−7.3	−3.9	1.10 (1.02, 1.23)

^a^ The uncertainty in the estimation of the binding parameters is reported as the confidence interval at a statistical significance of 95%, shown in parentheses below each parameter [68]. Association constant, *K*_a_; dissociation constant, *K*_d_; binding enthalpy, Δ*H*; binding stoichiometry (or percentage of binding-competent protein), n.

## Data Availability

The data and the vectors used are available from the corresponding authors upon reasonable request.

## Supplementary Materials

The following supporting information can be downloaded at https://www.mdpi.com/article/10.3390/cells11142166/s1: Figure S1. Binding to PADI4 of ΔImpα3 monitored by fluorescence; Figure S2. SEC chromatograms of NLS1/2-PADI4 in 20 mM Tris (pH 7.6) and 250 mM NaCl; Figure S3. The binding of both importin species to NLS1-PADI4 monitored by different spectroscopic probes; Figure S4. The binding as monitored by BLI (sensorgrams) of the NLS1-PADI4 and NLS2-PADI4 peptides; Figure S5. Affinity of the two NLSs of PADI4 predicted in docking simulations performed using a different template for Impα3; Figure S6. Binding location of seven -residue long fragments of the two NLSs of PADI4 predicted using a different template for Impα3; Figure S7. Location of the NLS regions Pro56–Ser83 and Arg495–Ile526 in the folded structure of PADI4; Table ST1. A reference list, and a table containing the ^1^H-NMR assignments of NLS2-PADI4.
