# Supplementary material for: Human Enzyme PADI4 Binds to the Nuclear Carrier Importin α3
Source: Cells. 2022 Jul 11;11(14):2166. doi: 10.3390/cells11142166 (PMC9319256; doi:10.3390/cells11142166)
Supplement: Supplementary file 1 [file cells-11-02166-s001.zip › cells-1761279-supplementary-update.pdf]

Supplementary Material

Human enzyme PADI4 binds to the nuclear carrier Importin α3

José L Neira, Bruno Rizzuti, Olga Abian, Salomé Araujo-Abad, Adrian Velazquez-Campoy and Camino de Juan Romero

FIGURE S1. **Binding to PADI4 of ΔImpα3 monitored by fluorescence.** (A) Fluorescence spectrum obtained by excitation at 280 nm of the PADI4/ΔImpα3 complex, and addition spectrum obtained by the sum of the spectra of the two isolated macromolecules. (B) Titration curve monitoring the relative changes of fluorescence at 330 nm when ΔImpα3 was added to PADI4. The fluorescence intensity on the y-axis is the relative fluorescence intensity after removal of the corresponding blank. The line through the data is the fitting to Eq. (1). Experiments were carried out at 25 ºC.

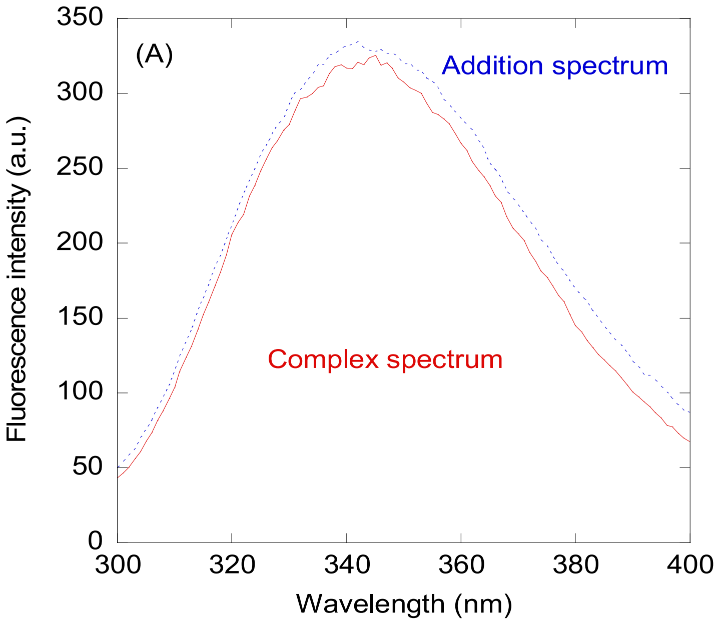

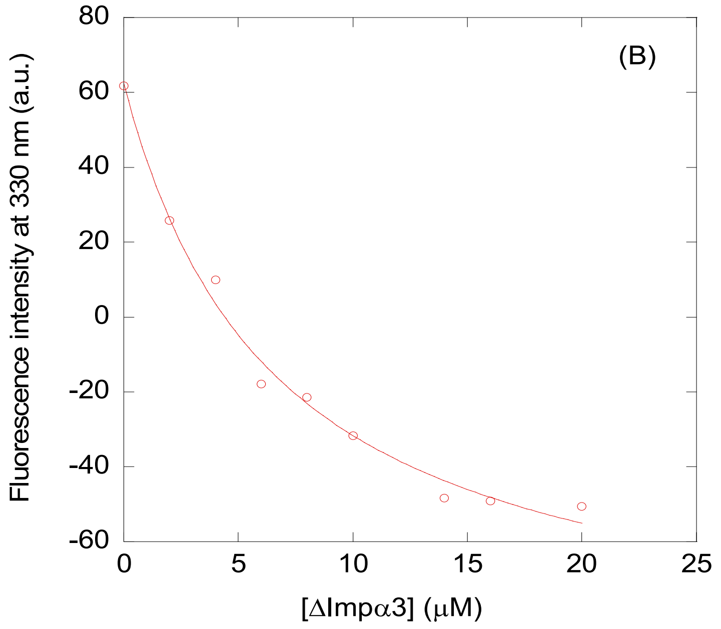

FIGURE S2. **SEC of NLS1/2-PADI4 peptides**: Chromatograms of both peptides in Tris 20 mM, pH 7.6 and 250 mM NaCl. The units of absorbance (at 280 nm) are arbitrary.

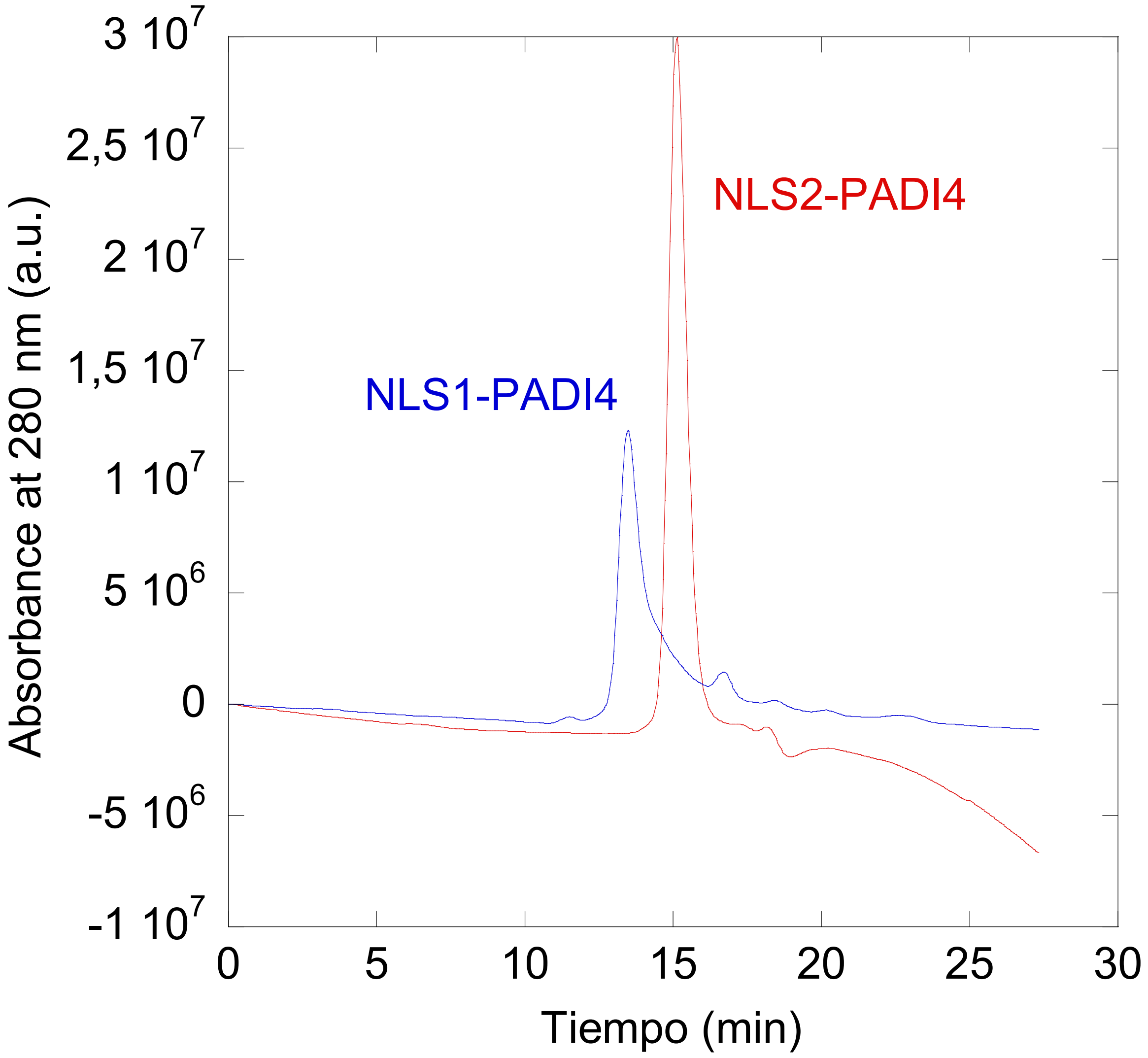

FIGURE S3. **Binding of importin species to NSL1-PADI4 as monitored by spectroscopic probes**: (A) Fluorescence spectrum of the complex Impα3/NSL1-PADI4 and that obtained by the addition of the spectra of the two isolated molecules. (B) Far-UV CD spectrum of the complex ΔImpα3/NSL1-PADI4 and that obtained by the addition of the spectra of the two isolated molecules. Experiments were carried out at 25 ºC.

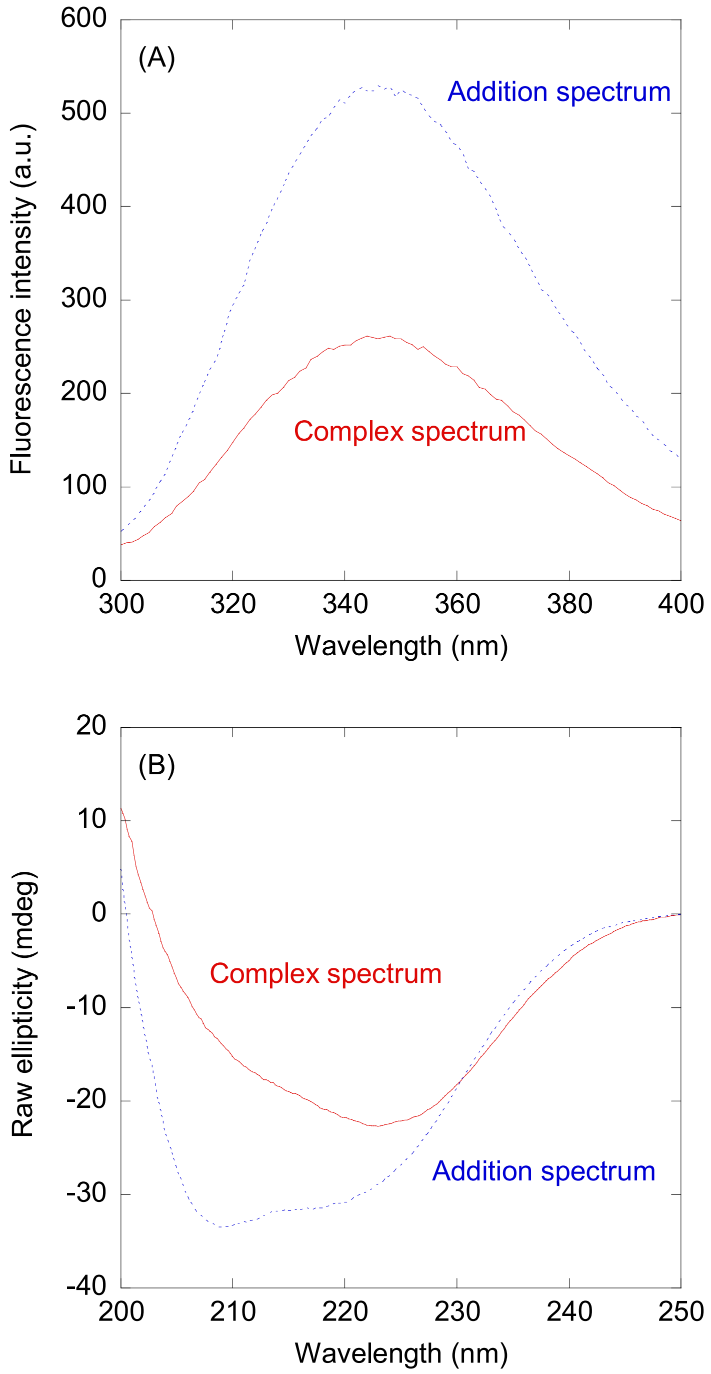

FIGURE S4. **Sensorgrams of NSL1-PADI4 monitored by BLI**: The curves correspond to the pseudo-first order plots shown in Fig. 7 for (A) Impα3 and (B) ΔImpα3. Experiments were carried out at 25 ºC.

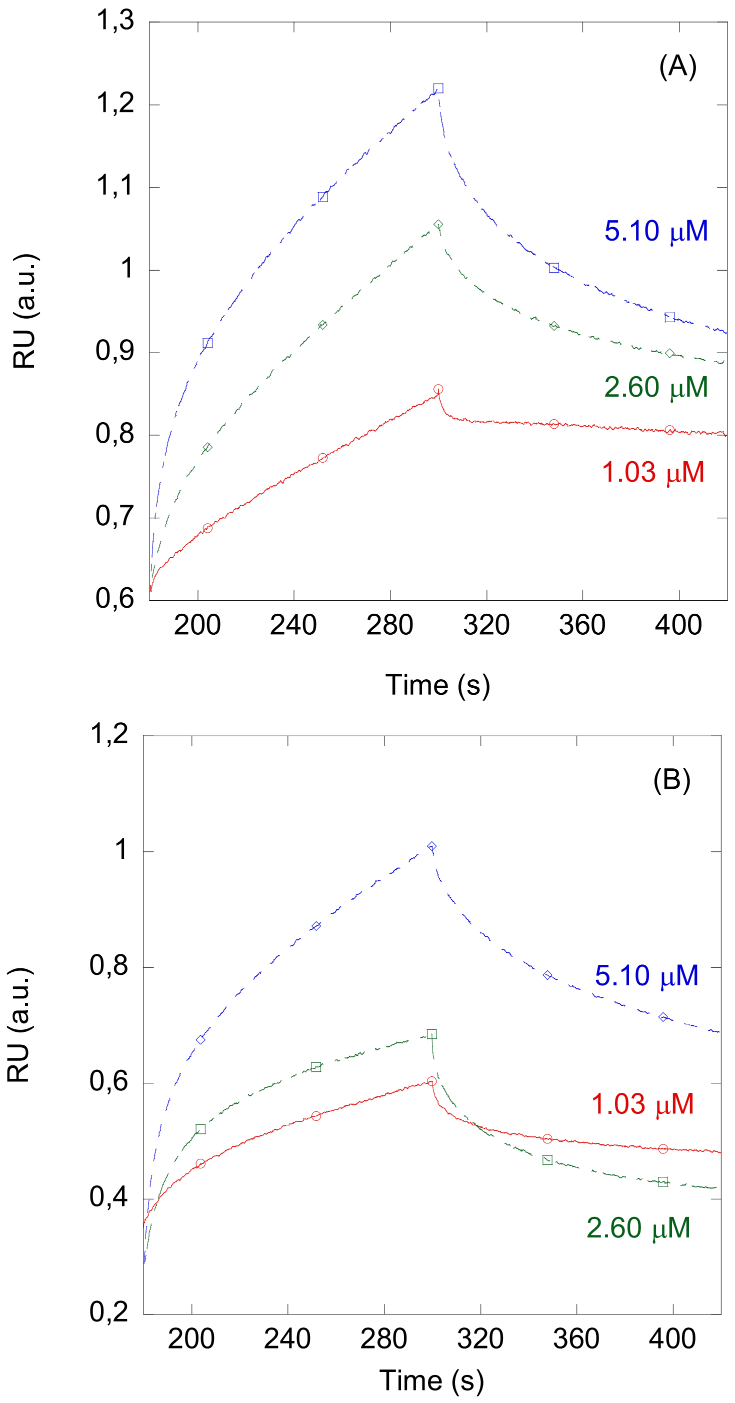

FIGURE S5. **Comparison of the affinity of the two NLS regions of PADI4 as estimated in molecular docking simulations performed by using different templates for Impα3.** The curves (black) obtained for the structure extracted from the PDB entry 5XZX ([65], in the main text), which reports the crystallographic complex of Impα3 with the NLS of the Ran-binding protein 3 (see also Fig. 10 in the manuscript) are compared with the curves (red) obtained for Impα3 structure built by using as a model the PDB entry 5X8N ([66], in the main text), which reports the crystallographic complex of Impα1 with the NLS of the Epstein-Barr virus EBNA-LP protein. (A) peptide NLS1-PADI4, encompassing residues 58-84; (B) NLS2 region of PADI4, including the N-terminal residues 490-497 and the peptide NLS2-PADI4 encompassing residues 498-526. The affinity is calculated for 7-residue-long fragments, and values reported for each residue are the average over all simulation runs including that residue.

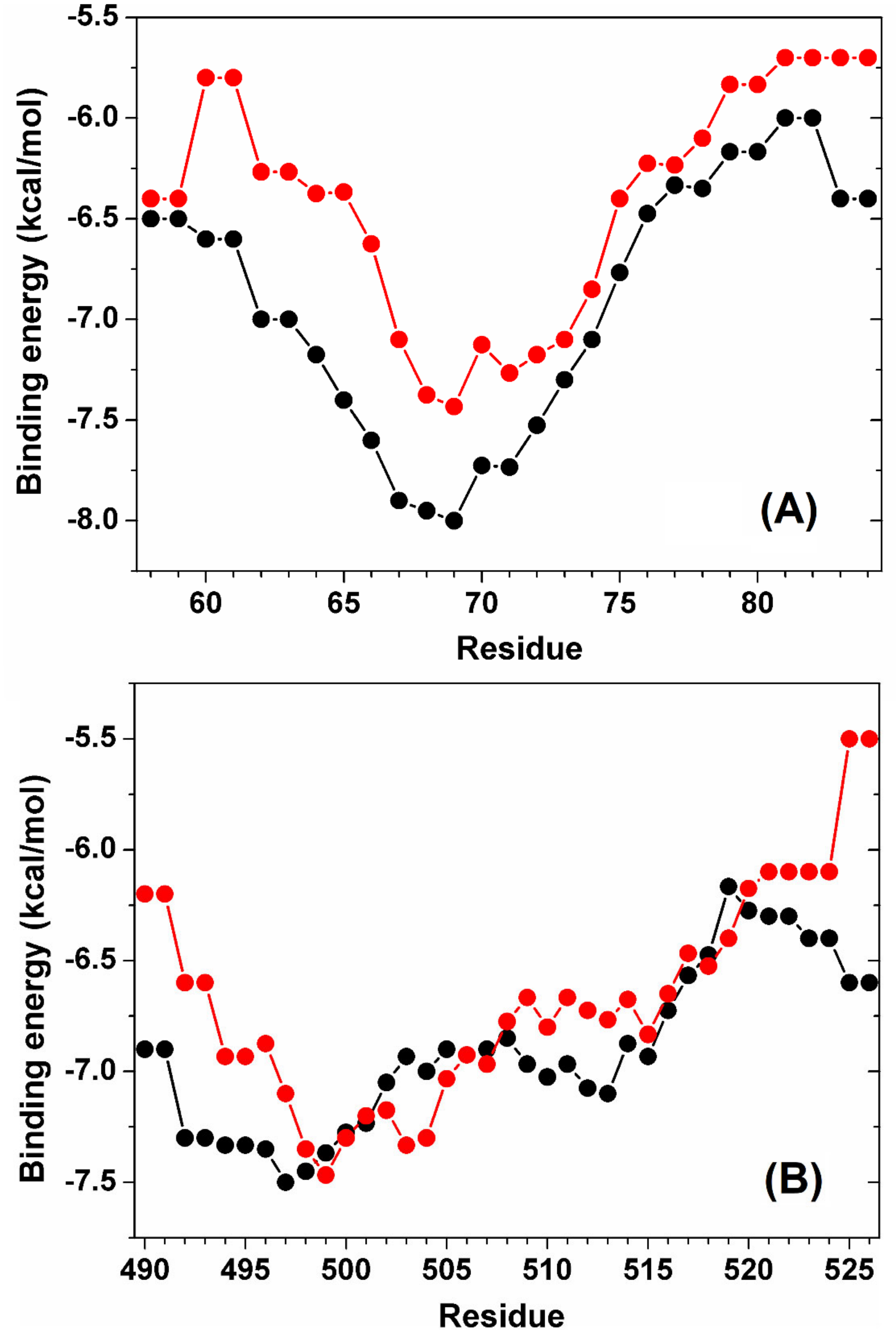

FIGURE S6. **Binding location of 7-residue-long fragments of the two NLS regions of PADI4 on the surface of Impα3 observed in simulations.** Results are shown for Impα3 structure built by using as a model the PDB entry 5X8N ([66] in the main text), which reports the crystallographic complex of Impα1 with the NLS of the Epstein-Barr virus EBNA-LP protein. (Left) conformations of peptide NLS1, and (right) peptide NLS2. Fragments follow the color scheme red → magenta → blue, going from the N to the C terminus of the two NLS peptides. (Center) comparison among the fragments with the most favourable binding affinity of (magenta) NLS1-PADI4 and (red) NLS2-PADI4, and (cyan) crystallographic pose of the NLS of the Epstein-Barr virus EBNA-LP protein. For clarity, the sole backbone chain is shown for all the sequences.

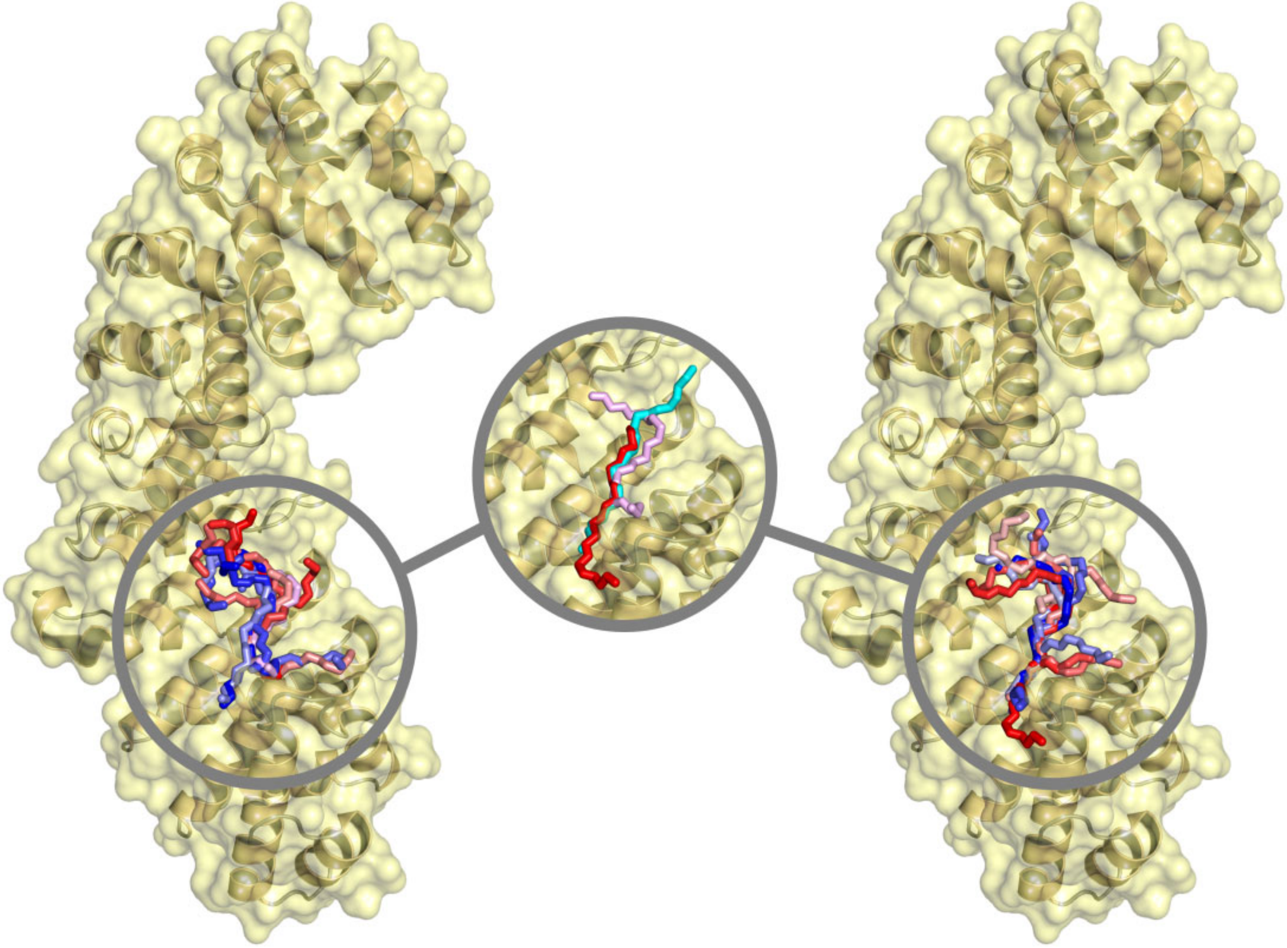

FIGURE S7: **Location of the two NLSs in the folded structure of PADI4**: The homodimer (shown either in van der Waals or ribbon representation) of PADI4 possesses in each monomer the two solvent-exposed NLS region 56-83 (blue) and 495-526 (red).

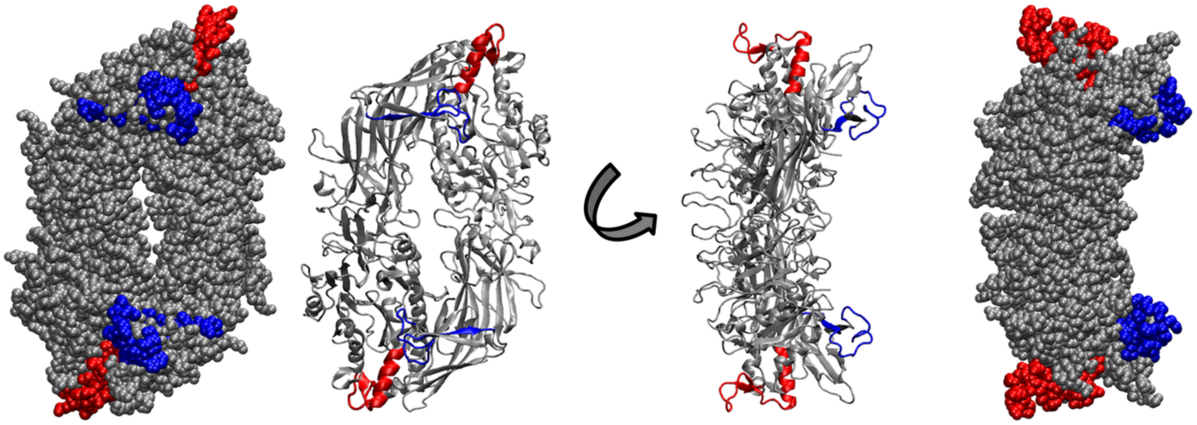

SUPPPORTING TABLE

Table S1. Chemical shifts (δ, ppm from TSP) of NLS2-PADI4 in aqueous solution (pH 7.0, 10 ºC)[a]

	NH	Hα	Hβ2	Hβ3	Hγ2	Hγ3	Hδ2	Hδ3	Hε	Hζ
Ac-Tyr498	8.29	4.48 (-0.09)	2.96				7.10		6.82	
Lys499	8.26	4.23 (0.03)	1.65		1.24					
Leu500	8.10	4.27 (0.05)	1.46				0.87 (Me)			
Phe501	8.28	4.55 (-0.11)	3.03				7.23		7.34	
Gln502*	8.53	4.25 (-0.05)	1.99		2.33					
Glu503*	8.50	4.23 (0.03)	2.00		2.30					
Gln504*	8.48	4.18 (-0.14)	1.95		2.28					
Gln505*	8.48	4.18 (-0.16)	1.95		2.28					
Asn506	8.56	4.69 (-0.01)	2.75							
Glu507	8.49	4.23 (-0.04)	1.95		2.30					
Gly508	8.53	3.96 (0.01)								
His509	8.52	4.76 (0.07)	3.17; 3.34							
Gly510	8.64	3.95 (-0.04)								
Glu511	8.60	4.25 (0.01)	1.93; 2.07		2.28					
Ala512	8.43	4.22 (-0.07)	1.38 (Me)							
Leu513	8.21	4.24 (-0.02)	1.59				0.89 (Me)			

Leu514	8.34	4.19 (-0.11)	1.78	1.45	0.87 (Me)	
Phe515	8.26	4.59 (-0.09)	3.03		7.24	7.34
Glu516*	8.53	4.25 (-0.01)	1.99	2.33		
Gly517	8.02	3.90 (-0.04)				
Ile518	8.02	4.09 (-0.04)	1.90	1.18; 1.50	0.89 (Me)	
Lys519*	8.44	4.22 (-0.09)	1.78			
Lys520*	8.51	4.29 (0.02)	1.76	1.39		
Lys521*	8.51	4.29 (0.01)	1.76	1.39		
Lys522*	8.51	4.29 (0.01)	1.76	1.39		
Gln523*	8.43	4.25 (-0.01)	1.95	2.28		
Gln524*	8.48	4.18 (-0.12)	1.95	2.28		
Lys525*	8.51	4.29 (-0.03)	1.76	1.39		
Ile526-Am	8.35	4.08 (-0.06)	1.78	1.45	0.91 (Me)	

[a] The (*) indicates those residues whose resonances could not be unambiguously assigned. The N terminus was acetylated and the C terminus was amidated. For the H_α proton column, the values within parenthesis are the conformational shifts (δ_{res}-δ_{rc}). The random-coil values for the sequence were obtained from: https://spin.niddk.nih.gov/bax/nmrserver/Poulsen_rc_CS/).
